# scPML: pathway-based multi-view learning for cell type annotation from single-cell RNA-seq data

**DOI:** 10.1038/s42003-023-05634-z

**Published:** 2023-12-14

**Authors:** Zhi-Hua Du, Wei-Lin Hu, Jian-Qiang Li, Xuequn Shang, Zhu-Hong You, Zhuang-zhuang Chen, Yu-An Huang

**Affiliations:** 1 College of Computer Science and Software Engineering, ShenZhen University, 3688 Nanhai Avenue, Shenzhen, China; 2 School of Computer Science, Northwestern Polytechnical University, Xi’an, China

**Keywords:** Computational models, Machine learning

## Abstract

Recent developments in single-cell technology have enabled the exploration of cellular heterogeneity at an unprecedented level, providing invaluable insights into various fields, including medicine and disease research. Cell type annotation is an essential step in its omics research. The mainstream approach is to utilize well-annotated single-cell data to supervised learning for cell type annotation of new singlecell data. However, existing methods lack good generalization and robustness in cell annotation tasks, partially due to difficulties in dealing with technical differences between datasets, as well as not considering the heterogeneous associations of genes in regulatory mechanism levels. Here, we propose the scPML model, which utilizes various gene signaling pathway data to partition the genetic features of cells, thus characterizing different interaction maps between cells. Extensive experiments demonstrate that scPML performs better in cell type annotation and detection of unknown cell types from different species, platforms, and tissues.

## Abstract (web summary)

scPML is a pathway-based multi-view learning model that outperforms alternative approaches in cell type annotation and detecting unknown cell types across diverse species, platforms, and tissues.

## Introduction

The technique of single-cell RNA sequencing (scRNA-seq) has revolutionized the analysis of cellular heterogeneity and opened up avenues for studying the mechanisms underlying development and disease at a single-cell resolution^1–3^. By contrast to bulk sequencing, which merely provides information on tissue averages^4^, scRNA-seq allows for transcriptomic studies on individual cells^5^. The crucial task of cell identification, which assumes a fundamental role in both biological and medical research, constitutes an indispensable component of scRNA-seq data analysis^6,7^.

Cell type annotation techniques, such as SCINA^8^, scSorter^9^, and Garnett^10^, commonly involve a two-step process that consists of unsupervised clustering of cells, followed by the assignment of cell types to each cluster based on the aggregated expression profiles of clustering results, as well as cross-referencing with sets of canonical gene markers. However, the accuracy of such annotations is heavily influenced by hyper-parameter settings (e.g., the number of clusters), and highly dependent on prior knowledge of canonical marker sets, which may be limited or unavailable for rare or less studied cell types^8,10^.

To address the challenges posed by marker genes, prevalent annotation methods now leverage cell-type-specific information from existing reference datasets^6,11–13^. These methods can be broadly categorized as correlation-based methods or machine-learning-based methods^14^. Correlation-based methods quantify the correlation of gene expression profiles between reference and query data. For instance, scmap^15^ projects cells from query data onto reference data and measures the correlation between them using high variable gens of reference data with three different correlation calculations (i.e., cosine similarity, Spearman correlation, and Pearson correlation). SingleR^16^ performs cell annotations in a similar fashion, while CHETAH^17^ constructs a classification tree based on the variance in gene expression profiles of each cell type in the reference data. These methods may be subject to batch effects, particularly when the reference and query data are derived from different platforms and experiments^18^. Although Seurat^19^ addresses batch correction by identifying anchor cell pairs between well-labeled reference data and unlabeled query data, accurately distinguishing biological perturbations along with technical batch effect^20,21^.

Machine learning-based methods, such as SciBet^22^, scNym^23^, are capable of recognizing cell-type-specific patterns by extracting key features of cells and assigning labels to query data. These methods are renowned for their ability to handle intrinsic noise and to overcome batch effects^20,22,23^. However, their performance remains restricted, partially due to their limitation in learning cell-type-specific patterns solely from individual cells while ignoring inter-cellular relationships. Numerous studies have demonstrated that Graph Convolutional Networks (GCN) can be utilized to capture such topological cell relationships and enhance performance^24–28^. The graph-based method scGCN^24^ employs the CCA-MNN approach to construct a hybrid graph consisting of both reference and query cells, thereby enabling scGGN to transfer labels from reference to query data. Machine learning methods that require the use of test data during the training process, such as scNym^23^ and scGCN^24^, are known as direct learning methods. For each new batch of test data, these methods need to be retrained with both the training and test data to annotate the new batch of test data, making them unsuitable for processing multiple batches of test data. Additionally, these methods do not consider the interactions between genes, which may weaken their performance in cell annotation tasks.

Identifying the sources of cell-to-cell variability in signaling dynamics is essential for cell annotation^29–33^. Here, we utilized different gene sets from biological pathways to partition cell gene features and constructed topological maps of cell-cell relations. We then employed graph convolutional neural networks (GCN)^34^ to capture high-order relationship information between cells and obtain low-dimensional representations^24,35^. Recently, pre-trained models have gained significant popularity. Geneformer^36^ is a pre-trained model based on self-attention mechanisms. It has undergone self-supervised learning on approximately 30 million cell data to gain an understanding of dynamic networks. After pre-training, Geneformer only requires context-specific fine-tuning and can be applied to various downstream tasks, such as network dynamics predictions and cell annotations.scArches^37^ is also a pre-trained algorithm that can be compiled with lots of different models, such as treeArchs^38^, which is used to construct a hierarchical tree from reference data to annotate cells of query data.Inspired by Geneformer and scArches, we designed a self-supervised GCN (Graph Convolutional Network) here to extract low-dimentional representations of raw data.

As there exist numerous pathway datasets^39–42^, we could construct many different cell topological maps from different perspectives on scRNA-seq data, each of which we term a “view." To fully exploit these distinct views, we used a multi-view learning approach^43,44^ to integrate these feature information. Consequently, we proposed scPML, an artificial intelligence neural network model based on graph convolutional neural networks and multi-view learning for annotating cell types. scPML simultaneously considers cell-cell relationships and gene-gene interactions with pathway and graph convolution network, respectively, and integrates information from different pathway datasets using multi-view learning. We have extensively demonstrated the superiority and robustness of scPML in annotating cell types from different platforms, species, and tissues through multiple experiments. Additionally, scPML can be conveniently applied in scenarios with multiple batches of test data without sharing training data, and pre-training can efficiently facilitate cumulative learning from multiple training data.

## Results

### Overview of scPML

**Fig. 1:**
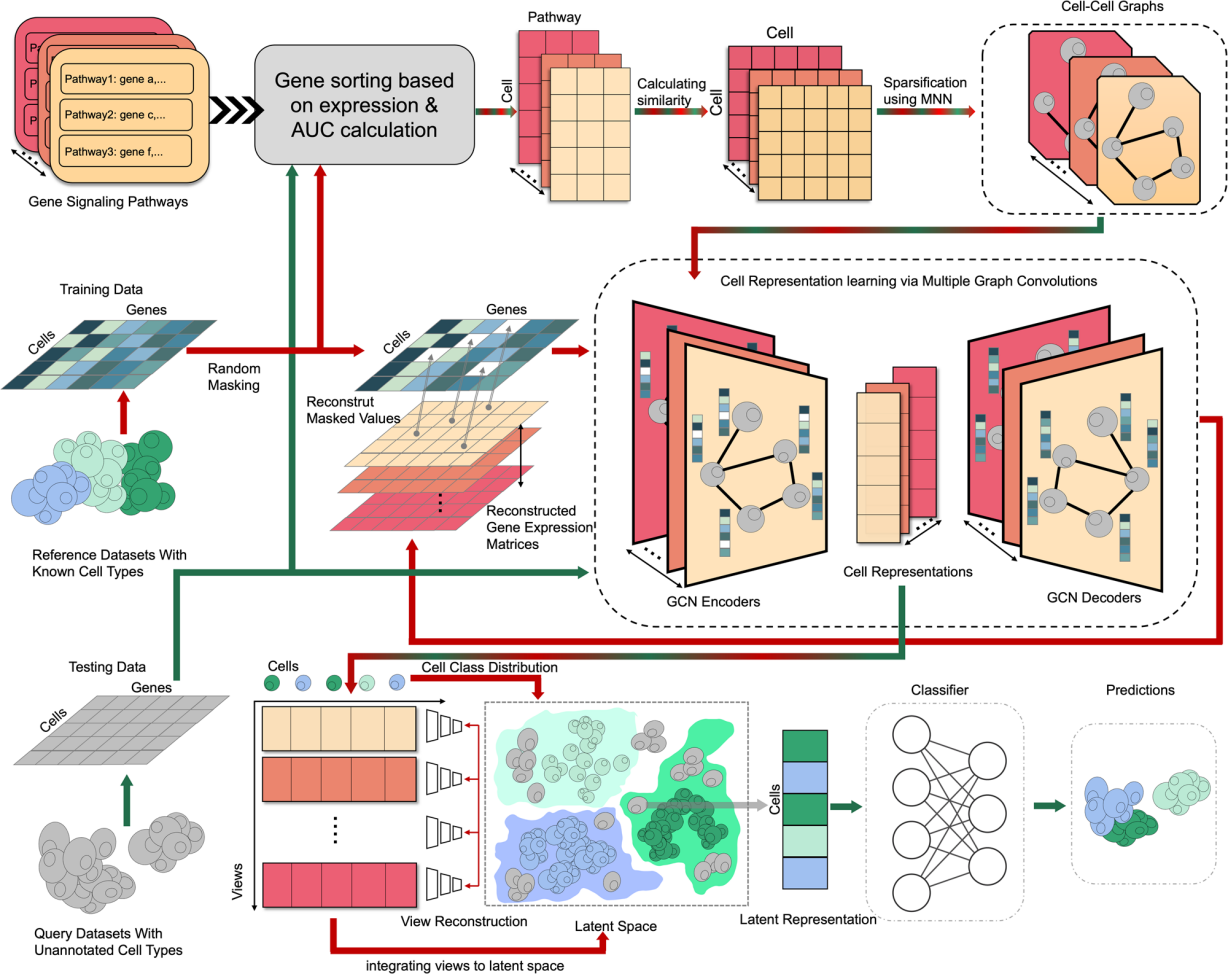
Overview of scPML. **a** scPML constructing cell-cell graphs using gene enrichment analysis with various pathways, yielding various cell-cell graphs marked with different colors. **b** Self-supervised GCN auto-encoder with the objective of recovering the masked units of processed expression data. The white grids are masked values which will be set to 0. **c** Obtaining common latent representation with multiple embeddings using multi-view learning. scPML attempts to find a common representation which can be reconstructed to according embeddings and has the quality of separability. After obtaining the common latent representations, scPML uses a classifier to assign labels.

The classification of a cell is predominantly determined by the genes it expresses, thus rendering gene expression data as an optimal basis for cell classification. scPML, utilizing well-labeled gene expression data, learns latent cell-type-specific patterns for annotating cells in test data (Fig. 1). scPML initially employs various pathway datasets to model multiple cell-cell graphs to learn kinds of relationships among cells for a training dataset. Pathway datasets divide genes into various gene sets based on specific biological processes^39^, which reflect cell heterogeneity on the level of biological functions and minimize the impact of dropout events as a gene has limited effect on the entire gene set^29^. We use pathways to construct a similarity matrix, which reflect the similarity between cell. Then we use mutual nearest neighbor (MNN)^18^ concept to construct cell-cell graphs. Structural information is learned from cell-cell graphs using self-supervised convolutional neural networks in scPML to produce denoised low-dimensional representations for cells. Traditional auto-encoders can reduce the dimensions and denoise the features^24,45,46^, but they disregard the high-order relations between cells. Injecting auto-encoders into GCN can capture the structural information of the data while reducing the dimension. It is noteworthy that unlike conventional auto-encoders, some non-zero gene expression values are masked before training, and scPML reconstructs them through the GCN-based auto-encoders, effectively minimizing the impact of dropout events. Different pathways can describe the training data from distinct perspectives, which may complement each other^43^. To utilize this knowledge sufficiently, multiple independent GCNs are used to extract representations from various views, followed by the use of multi-view learning to integrate them and obtain a shared latent subspace representation^43^. Lastly, a classifier is employed to assign cell types by learning cell-type-specific patterns from latent representations. For test data, it is pre-processed, and graphs are constructed in the same fashion. The parameters optimized for scPML are utilized to assign cell types in test data. Unlike most semi-supervised models, scPML does not require knowledge of test data during training and can still capture essential features and generalize the learned patterns to new data regardless of batch effects.

### Cell type annotation across platforms

**Fig. 2:**
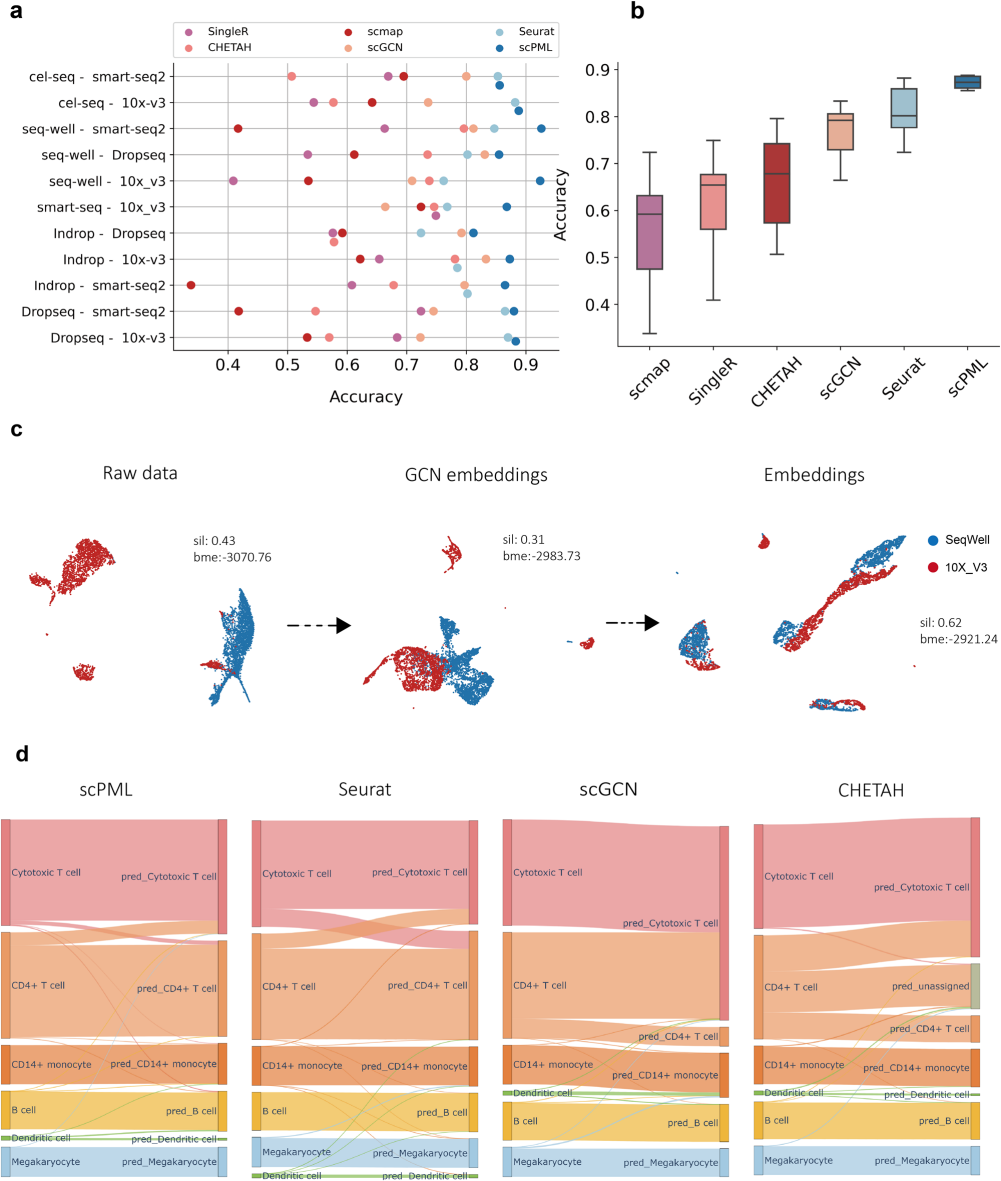
Comparison of scPML with other methods in cross-platform experiments. **a** The performance of scPML and other methods is measured by accuracy score for 11 training-test data pairs, where training and test data are profiled using different scRNA-sequence protocols. The y-axis represents each experiment, and the x-axis stands for accuracy. Each point corresponds to the accuracy of a method in an experiment. **b** Box plots are used to illustrate the accuracy results of all methods, where the middle line represents the median, the lower and upper hinges represent the first and third quartiles, and the whiskers extend to the range of 1.5 times the interquartile range (IQR). **c** The UMAP projections of cells from SeqWell-10X V3 with features of raw data and latent representations produced by self-supervised graph convolutional layer, as well as embeddings from the classifier of scPML are presented. Silhouette score and batch mixing entropy are abbreviated as sil and bme respectively. **d** Sankey plots of scPML, Seurat, scGCN, and CHETAH for SeqWell-10X V3 are shown, where the left column represents the true labels of cells, and the right column represents the predictions.

The rapid development of single-cell sequencing technology has led to the generation of vast amounts of single-cell datasets from diverse experiments and sequencing platforms. However, batch effects can make it challenging to accurately annotate cell types (Fig. 2c, d). In this study, we evaluated the ability of scPML to annotate cell types for cross-platform experiment.

We used 12 well-labeled Peripheral Blood Mononuclear Cells (PBMCs) datasets from six distinct sequencing platforms^47^, and for each pair of training-test dataset generated by different sequencing technologies, we utilized one dataset as training data to predict the cells in the other one as test data. We compared scPML’s performance with other methods, including Seurat^19^, scmap^15^, CHETAH^17^, SingleR^16^, scGCN^24^, Geneformer^36^ and scArches^37^, using accuracy score and Macro F1 (Supplementary Fig. 8) as the evaluation metrics. Our results consistently showed that scPML outperformed other methods (Fig. 2a, Supplementary Fig. 12b), with an accuracy of 0.87 compared to Seurat (mean accuracy of 0.81), scGCN (mean accuracy of 0.78), Geneformer (mean accuracy of 0.72), CHETAH (mean accuracy of 0.70), scmap (mean accuracy of 0.700), scArches (mean accuracy of 0.65) and SingleR (mean accuracy of 0.619). The Macro F1 also showed the superior performance of scPML (Supplementary Fig. 8). Correlation-based methods such as CHETAH, scmap and SingleR have been shown to exhibit lower accuracy partially due to their limited capacity to handle batch effects. In contrast, scPML demonstrates superior performance in cross-platform experiments, indicating its ability to recognize cell-type-specific patterns regardless of batch effects. To further support this claim, we visualize the latent representations generated by the GCN layer and classification layer of scPML, and we can see that the self-supervised GCN layer can effectively alleviate batch effects (Fig. 2c) with a higher batch mixing score of -2883.72 than raw data (batch mixing entropy = −3070.75). Furthermore, UMAP^48^ projections of the embedding from the classifier demonstrate that the training and test data are well mixed and primarily grouped by their cell labels (Supplementary Fig. 1), providing evidence that the self-supervised GCN module of scPML is capable of capturing low-dimensional representations of the training and test data, thereby alleviating batch effects and leading to improved prediction. To ensure experimental fairness, we also conducted reversed cross-platform experiments (Supplementary Note 4 and Supplementary Fig. 15). The results similarly demonstrate the superior performance of scPML.

The Sankey plots for all methods applied to SeqWell-10X V3 is presented (Fig. 2d, Supplementary Fig. 2). scmap assigns most CD14+ monocyte cells to Dendritic cells, while SingleR incorrectly assigns some B cells to Dendritic cells. Seurat and scGCN are able to accurately classify most cells. However, they can not clearly distinguish CD4+ T cells and Cytotoxic T cells due to their high similarity. In contrast, scPML outperforms the other methods in distinguishing CD4+ T cells and Cytotoxic T cells. Although the anchors of Seurat and scGCN can be utilized to correct batch effects, it is believed that they sometimes distort biological signals along with technical perturbations, particularly in cases where cells are closely related. To further explore this, the anchors of Seurat and scGCN for SeqWell-10X V3 were examined. For Seurat, only 68 out of 325 anchors in CD4+ T cells (average quality score of 0.248) were paired with CD4+ T cells with an average quality score of 0.244, while 111 anchors were mis-paired with Cytotoxic T cells with an average quality score of 0.264. For scGCN, out of 2545 anchors in CD4+ T cells, only 544 anchors were paired with CD4+ T cells, while 1674 anchors were paired with Cytotoxic T cells. It is maintained that Seurat and scGCN may distort biological signals when modeling anchors between reference and query datasets alongside batch effects.

It is worth noting that scPML exhibits a remarkable ability to accurately classify Dendritic and Megakaryocyte cells, despite their infrequent occurrence in the datasets (Fig. 2d), thus highlighting the robustness of scPML to imbalanced class distributions. Moreover, scPML demonstrates a stable and consistent performance for cross-platform prediction, as depicted in Fig. 2b. In contrast, other methods exhibit significant variations in their performance across different training-test pairs, while scPML shows only a slight variation, suggesting the generalizability of scPML for cross-platform annotation tasks.

### Cell type annotation across species

By annotating cell types across species, researchers can develop more detailed phylogenies of cell types that can help to understand the evolutionary and developmental connections between cell types in different species^49–51^. In this study, we aim to annotate cells for a particular species, such as humans, by using cells from another species, such as mice, as a training set. This presents a challenge, but one that we are eager to take on. To accomplish this, we have designed four experiments, each consisting of a training-test pair that uses cells from the mouse and human pancreas. In total, we are examining 11 cell types, including the major cell types of the pancreas (alpha, beta, delta, and gamma cells) with with Baron:mouse^52^, Baron:human^52^, Xin^53^, Muraro^54^, and Segerstolpe^55^. We combined Xin, Muraro, Segerstolpe and Baron:human as Combination (human). To extract the most relevant features for cell annotation, we focus on the common homologous genes between the training and test data.

**Fig. 3:**
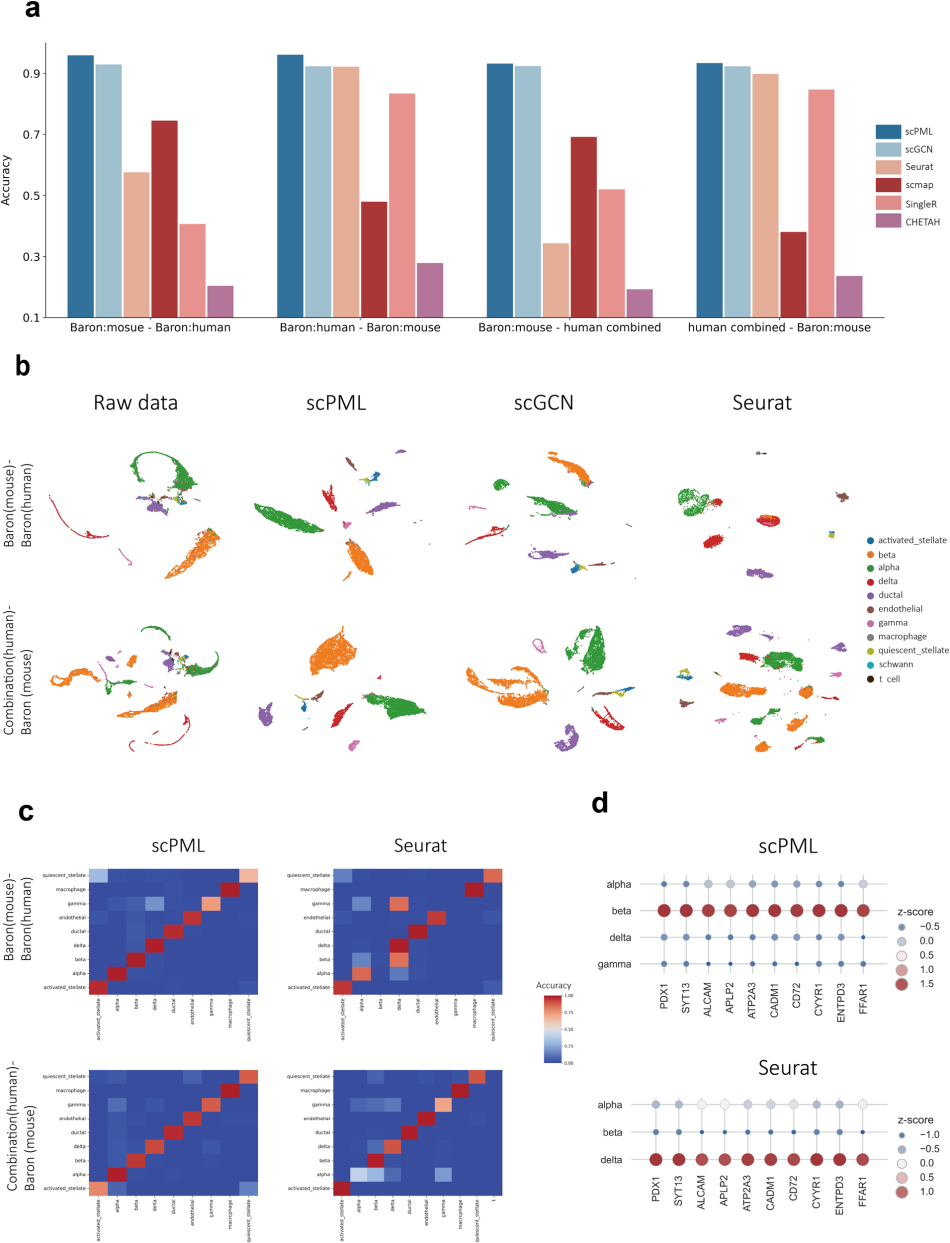
Comparison of scPML with other methods on mouse and human pancreas datasets when training and test data are from different species. **a** The performance of scPML and other methods (scGCN, Seurat, SingleR, scmap, and CHETAH) is measured by accuracy score for 4 paired cross-species datasets, and bar plots are used to illustrate the results. **b** UMAP projections of Baron (mouse)-Baron (human) and Combination (human)-Baron (mouse) by raw data and different methods (scPML, scGCN, Seurat) are presented, where 11 cell types are displayed. The first row represents Baron (mouse)-Baron (human), and the second row represents Combination (human)-Baron (mouse). **c** Confusion matrices of different methods (scPML, scGCN, Seurat, and scmap) for Baron (mouse)-Combination (human) are shown, where 9 cell types are displayed. The rows represent true labels, while the columns represent predicted labels. **d** Dot plots of marker gene expression for alpha and beta for cells with labels obtained from scPML and Seurat for the Baron (mouse)-Baron (human) dataset are presented. The beta marker genes were selected from CellMaker^62^.

For cross-species annotation, the scPML algorithm exhibited the best performance, as evidenced by its average accuracy of 0.94 (Fig. 3a, Supplementary Fig. 12a). This value is significantly higher than that of Seruat (mean accuracy of 0.88), Geneformer (mean accuracy of 0.81) scmap (mean accuracy of 0.807), SingleR (mean accuracy of 0.655), scArches (mean accuracy of 0.54) and also superior to scGCN (mean accuracy of 0.927). Conversely, CHETAH demonstrates a low accuracy of 0.231, indicating its inability to recognize shared genome patterns across different species. Of note, scPML also achieves a high accuracy of 0.94 when applied to the human-mouse paired dataset, suggesting its robustness to batch effects in training data. In addition, the Macro F1 also showed the superior performance of scPML in cross-speices experiments (Supplementary Figs. 7 and 12).

To provide a more intuitive representation of scPML’s annotation results, we compared the UMAP projections of cells using different methods with the training-test pair data (Fig. 3b, Supplementary Fig. 3). The raw data displays inadequate separation of cell clusters due to noise and batch effects, particularly for the paired dataset Combination (human)-Baron (mouse), where the alpha and beta cells are distributed into multiple clusters and ductal cells are intermixed with other cells. Seurat fails to separate most cells, such as alpha, beta, and delta cells, which is further evidenced by the confusion matrix (Fig. 3c, Supplementary Fig. 4). We further verify the the results by selecting marker genes for beta cells and displaying gene expression dot plots for labels predicted by Seurat and scPML (Fig. 3d). Known marker genes for beta cells have high expression for scPML-predicted beta cluster. In constrst, known marker genes for beta cells have high expression in alpha cluter predicted by Seurat. Although scGCN is able to discriminate most cells, the adjusted rand index (ARI) and Silhouette score indicate that scGCN’s clustering results are inferior to scPML (Supplementary Fig. 5, Supplementary Fig. 6). Conversely, scPML is able to clearly discern cells of different types and achieves superior performance in clustering. Notably, scPML overcomes batch effects in the training data in Combination (human)-Baron (mouse), resulting in well-separated cell subpopulations and further confirming its robustness to batch effects in training data. Collectively, these results suggest the excellent and robust performance of scPML for cross-species annotation.

### Benefits of multi-view learning

In practical scenarios, objects are often described from multiple perspectives, such as utilizing multiple types of features. For instance, an image can be identified by considering its color and texture features. Empirical studies have shown that leveraging multiple views can complement each other and improve performance^43^. For single-cell annotations, we model multiple cell-cell graphs of single-cell data using various pathways, which can partition genes into various subsets based on distinct biological processes. These multiple graphs can be regarded as different views for the single-cell data. Through the aggregation of GCN layers, we can generate multiple low-dimensional representations for each cell, which can be integrated using multi-view learning methods. We designed experiments to showcase the advantages of multi-view learning. We selected four training-test data pairs from cross-platform and cross-species experiments, including Baron (mouse)-Baron (human), Baron (human)-Baron (mouse), SeqWell-10X V3, and SeqWell-SmartSeq. Given four views produced from multiple pathways (KEGG^39^, Reactome^40^, WikiPathways^41^, yan^42^), we tested all possible combinations of views within the range of view numbers from 1 to 4.

**Fig. 4:**
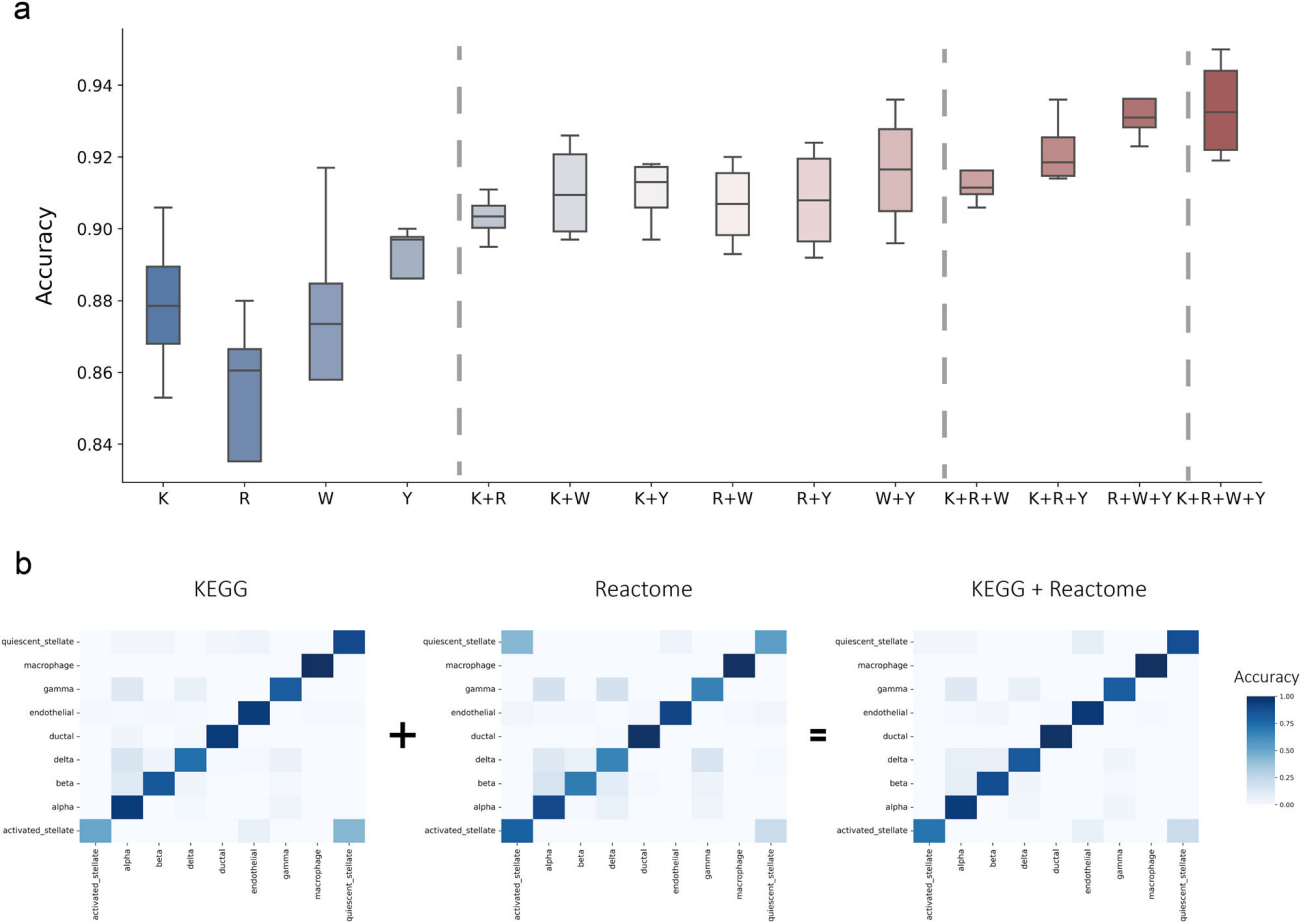
Evaluation of the performance of different views on paired datasets. **a** The boxplots illustrate the accuracy scores of different views on four paired datasets, with the gray dotted line separating the four groups representing different numbers of views. The views are represented by KEGG, Reactome, WikiPathway, and Yan with abbreviations K, R, W, and Y, respectively. In the box plot, the middle line represents the median, the lower and upper hinges represent the first and third quartiles, and the whiskers extend to the range of 1.5 times the interquartile range (IQR). **b** The heatmap displays the confusion matrices for single-view (KEGG, Reactome) and two-view (KEGG + Reactome) models on the Baron (mouse)-Baron (human) dataset. Rows correspond to true labels, while columns represent predicted labels.

We have conducted experiments to investigate the effectiveness of scPML in multi-view learning for single-cell annotation. Our study includes four cases, namely, single-view, two-view, three-view, and four-view, with all possible combinations of views. Our results reveal that multi-view learning generally outperforms single-view learning in terms of accuracy, indicating the benefits of integrating multiple views (Fig. 4a). Interestingly, our experiments show that increasing the number of views generally leads to better performance, as indicated by the rising trend in accuracy (Fig. 4a). Specifically, in the Baron (mouse)-Baron (human) dataset, the four-view case achieves the highest accuracy of 0.951, followed by the three-view case (mean acc = 0.938), two-view case (mean acc = 0.919), and single-view case (mean acc = 0.877).The Macro F1 also demonstrates the advantages brought by multiple views (Supplementary Fig. 9). However, we also observed that too many views may not always yield the best performance, as demonstrated by the case of SeWell-10x V3, where a three-view case of Reacome + WikiPathway + Yan (i.e., R+W+Y) performs better with higher accuracy (acc = 0.924) than the four-view case (acc = 0.915).

Multiple views can enhance the performance of cell annotations due to the complementary nature of different views, where each view may provide knowledge that other views may lack^43^. To gain a deeper understanding of why multi-view learning outperforms single-view learning, we utilized confusion matrix to visualize the accuracy of each cell type. Our results, as shown in Fig. 4b, indicate that in the Baron (mouse)-Baron (human) dataset, KEGG misclassifies most activated stellate cells to quiescent stellate cells, while Reactome misclassifies most quiescent stellate cells to activated stellate cells. This suggests that each pathway contains unique knowledge that the other pathway lacks. However, by combining KEGG and Reactome, the multi-view learning approach can integrate these knowledge sources, leading to more accurate cell type predictions, as evidenced by the correct prediction of most activated stellate cells and quiescent stellate cells in the right part of Fig. 3b. In summary, these empirical results suggest that multi-view learning can significantly improve cell annotation accuracy by integrating complementary knowledge sources.

### Multiple training data

In some scenarios, the lack of sufficient cells for certain cell types in the training data can result in poor performance of cell annotation. A straightforward solution is to collect more training data and combine them to obtain a larger training data, as more data can provide more knowledge and lead to better performance^20,56^. However, there are two major challenges in this approach. Firstly, combining multiple training data can inevitably introduce batch effects. Secondly, the combined data may require a large amount of memory, and retraining the model with the combined data is computationally expensive. It is noteworthy that scPML can effectively address the first challenge. The superior performance of scPML in the Combination (human)- Baron (mouse) case in cross-species experiments suggests the robustness of scPML to batch effects in combined training data. For the second challenge, we employed pre-training, which has proven effective in other applications^36–38^. Owing to the neural network and supervised learning method of scPML, we can obtain a well-trained scPML model using a small amount of training data. When new training data become available, we can retrain the model with the new data based on the existing parameters to obtain more knowledge. Due to the semi-supervised learning method, other methods such as scGCN and Seurat can only manually combine training data to handle inadequate data situations.

We designed two experiments to examine whether scPML can achieve a rising trend in accuracy with more training data. In the first experiment (PBMCs), we used SeqWell, DropSeq, and Indrop as training data and 10X V2 as test data. In the second experiment (MCA liver)^57^, we used three different training data sets sampled from different ages of mice (eight months, neonatal, ten days) as training data and adult liver cells as test data.

**Fig. 5:**
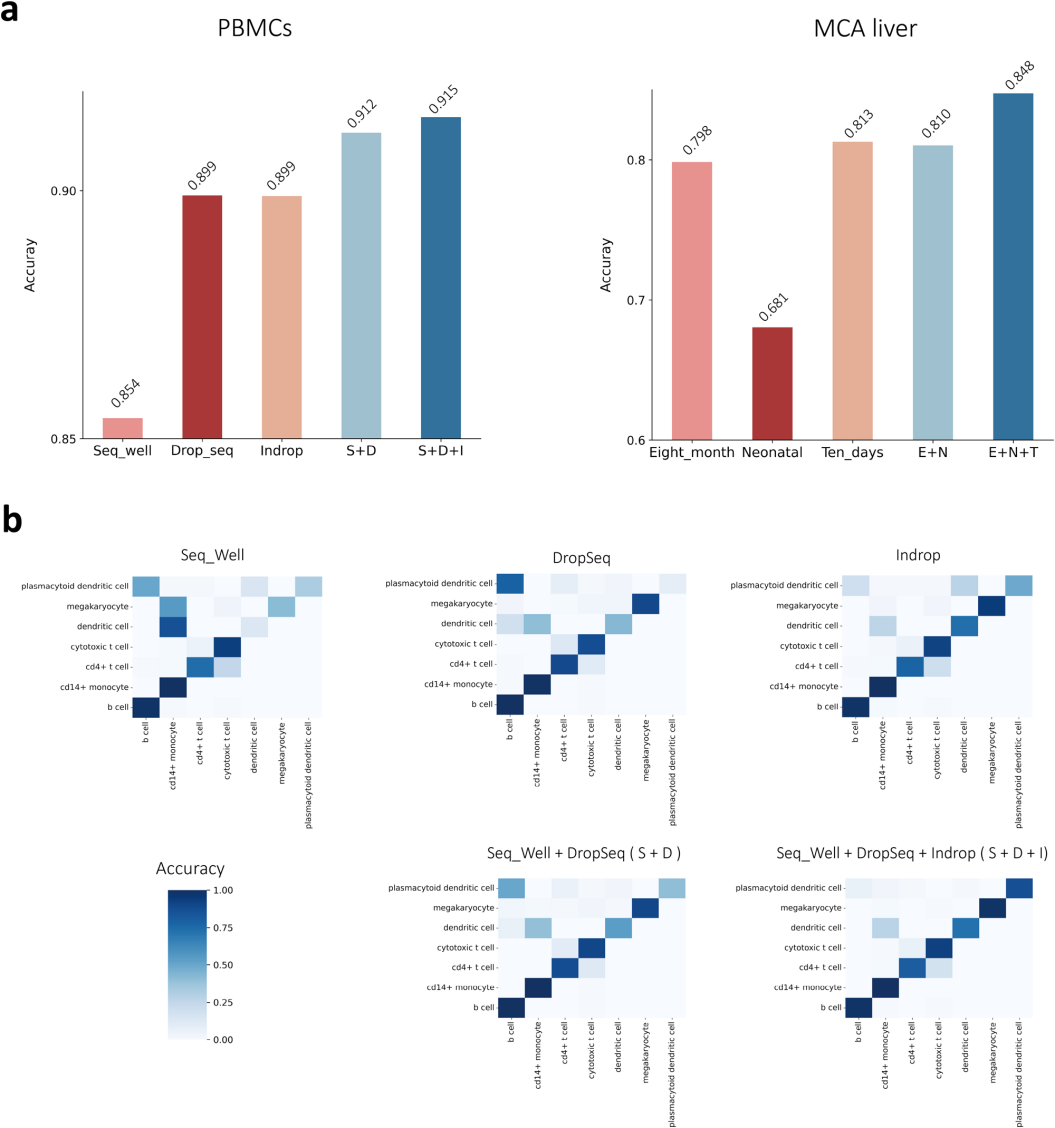
Multiple training data experiments. **a** The accuracy scores of scPML on PBMCs and MCA liver datasets are shown using bar plots with different multiple training data settings. **b** Heatmaps of confusion matrices are shown for single training data methods (SeqWell, DropSeq, and Indrop) and multiple training data methods (SeqWell+DropSeq and SeqWell+DropSeq+Indrop) using pre-training. Rows represent the true labels of cells, while columns represent the predicted labels.

In this study, we evaluated the performance of scPML on single and multiple training data sets in PBMCs and MCA liver. For multiple training data sets, we employed a pre-trained model and fed it with the new training data. For instance, in the SeqWell+DropSeq (S+D) case, we utilized DropSeq to feed the model, which had already been trained on SeqWell data. The same procedure was applied to the SeqWell+DropSeq+Indrop (S+D+I) and MCA liver experiments. The results showed that the S+D data set achieved an accuracy of 0.912 (Fig. 5a), which was higher than the DropSeq data set (acc = 0.899), and the E+N data set (acc = 0.810) had a higher accuracy than the Neonatal data set (acc = 0.681)). Additionally, S+D+I had a better performance than InDrop, and E+N+T had better performance than Ten days in the MCA liver case. These findings indicate that pre-training can improve the performance of scPML. Further analysis revealed that scPML is capable of accumulating knowledge from multiple data sets through pre-training. When we provided more training data, the accuracy of both PBMCs and MCA liver showed an upward trend. Specifically, the accuracy of SeqWell was 0.854, and scPML achieved a higher accuracy of 0.912 with S+D. Furthermore, when provided with InDrop data, the accuracy of scPML increased to 0.915 with S+D+I. Similar observations were made in the MCA liver case, where the accuracy of Eight month was 0.798, E+N was 0.810, and E+N+T was 0.848 (More details can be seen in Supplementary Figs. 10 and 11, including Macro F1 and cases of S+D, S+I and E+T, N+T). Overall, these results suggest that pre-training can enhance cell annotations by enabling scPML to learn multiple data sets cumulatively.

To further elucidate the advantages of pre-training, we conducted a heatmap analysis to visualize the accuracy of each cell type. As illustrated in Fig. 5b, when using only DropSeq data, scPML erroneously assigns most Plasmacytoid dendritic cells to the B cell category. However, the scPML model trained on SeqWell data and then fed with DropSeq data in the S+D case retains the ability to distinguish between Plasmacytoid dendritic cells and B cells. Furthermore, from the perspective of SeqWell data, the incorporation of DropSeq data enhances scPML’s ability to predict Megakaryocytes and Dendritic cells, presumably due to the acquisition of additional knowledge from DropSeq. Notably, the S+D+I approach outperforms S+D, SeqWell, DropSeq, and InDrop in classifying each cell type. Collectively, our results demonstrate that scPML can leverage pre-training to accumulate knowledge from multiple datasets and achieve improved performance without the need for data integration.

### Identification of unknown cell types

In certain practical applications, testing datasets may contain unknown cell types that are not represented in the training data, making it essential for cell annotation methods to identify these unknown cells. Marker gene-based methods may face challenges in differentiating unknown cells due to the absence of prior knowledge. Conversely, neural network-based methods can automatically identify unknown cell types by evaluating the predicted probability. Similarly, some correlation-based methods, such as CHETAH and scmap, support the identification of unknown cells by assessing the confidence score.

To effectively detect unknown cells, an ideal method should not only distinguish between known and unknown cells by producing low confidence scores for the latter, but also accurately identify each known cell type. In order to evaluate the performance of scPML in detecting unknown cells, we compare its performance with that of other methods, including scGCN, CHETAH, and scmap, using the Macro F1 score in binary classification scenarios where cell types are considered either known or unknown. A higher Macro F1 indicates better performance in distinguishing between known and unknown cells. Furthermore, we use accuracy score to assess the ability of each method to classify known cells. We apply all methods to three paired tumor datasets (GSE72056-GSE103322, GSE103322-GSE72056, GSE118056-GSE117988), where malignant cells are excluded from the training data but retained and marked as “unknown" in the test data. For machine-learning based methods (scGCN and scPML), cells with probability of model prediction lower than 0.5 for all known cell types are manually annotated as ‘unknown’.

**Fig. 6:**
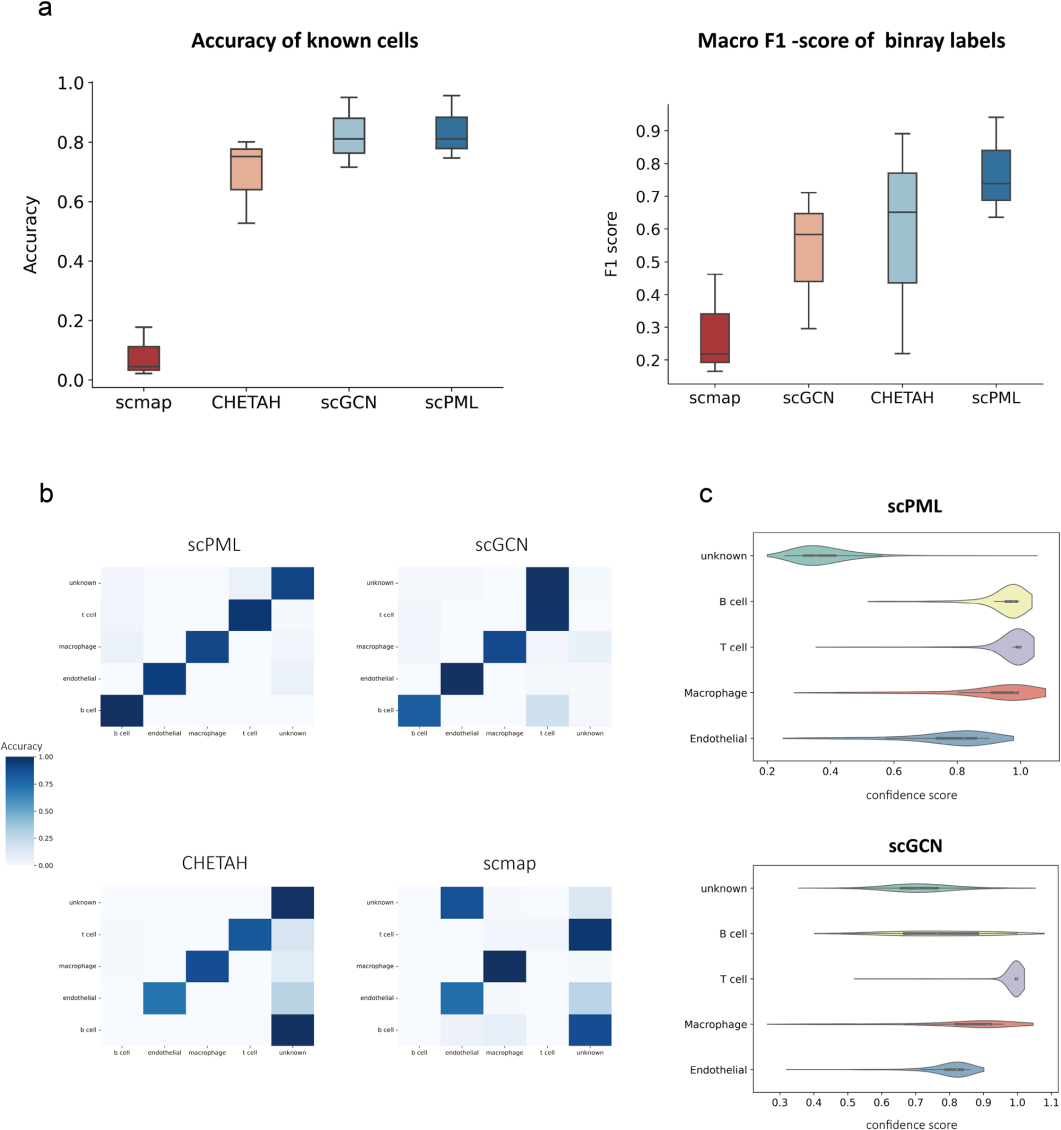
Comparison of the Performance of scPML and Three Existing Methods. **a** Boxplots show the accuracy of classifying known cells and Macro F1 score of binary classification with known and unknown cell labels for scPML, Seurat, scGCN, and scmap. In the box plot, the middle line represents the median, the lower and upper hinges represent the first and third quartiles, and the whiskers extend to the range of 1.5 times the interquartile range (IQR). **b** Heatmap illustrates the correlation between true labels and predicted results for scPML, Seurat, scGCN, and scmap. **c** Violinplots display the confidence scores provided by scPML and scGCN for GSE72056-GSE103322 data pair, where the white dot represents the median, the left and right hinges in the black area represent the first and third quartiles, the whiskers extend to the range of 1.5 times the interquartile range (IQR), the shape displays the distribution of data and the width of the plot at a given point represents the estimated density of the data at that value. Overall, scPML demonstrates superior performance in single-cell RNA sequencing analysis compared to the other methods evaluated in this study.

scPML exhibits superior performance compared to other methods in terms of Macro F1 (Fig. 6a), achieving an average of 0.807, a substantial margin over CHETAH (0.587), scGCN (0.53), and scmap (0.282), which suggests its capacity to accurately identify malignant cells. It should be noted that CHETAH shows a slight advantage over scGCN in detecting unknown cells (Fig. 6a). In the classification of known cells, scPML attains a mean accuracy of 0.836, higher than scGCN (0.826), CHETAH (0.693), and scmap (0.08), indicating its superior ability to categorize cells with known types. To further illustrate these findings, we employ confustion matrix to present the predictions for each cell of all methods (Fig. 6b). While scGCN is capable of classifying most known cells, it struggles to detect unknown cells by categorizing most of them as T cells. On the other hand, CHETAH discerned unknown cells from known cells but incorrectly assigned most B cells to the unknown type. In contrast, scPML demonstrated a remarkable ability to accurately distinguish between each cell type by generating low confidence scores for unknown cells and high confidence scores for cells with known cell types (Fig. 6c). Overall, these results suggest that scPML possesses the capacity to accurately detect unknown cells that were not present in the training data while also providing accurate predictions for known cells.

## Discussion

Single-cell sequencing technology enables the study of cellular heterogeneity at the level of individual cells and provides insights into the differentiation and development processes of cells. Annotation of single-cell types is a crucial step in scRNA-seq analysis, which helps researchers investigate the role and mechanisms of different cell types in disease occurrence and development, thereby aiding in disease diagnosis and treatment. In single-cell type annotation, the relationships between cells and genes are two important pieces of information that, if fully utilized, can help reduce noise and batch effects in scRNA-seq data. Therefore, we propose scPML, a supervised cell type prediction model that fully utilizes gene relationships in pathways to model single-cell networks from multiple perspectives. We collected 14 datasets and designed 17 experiments, including the detection of data from different platforms, species, and unknown cell types, using evaluation metrics such as Accuracy, F1-score (Macro), ARI, Silhouette, and Confusion matrix. We demonstrated that scPML outperforms other methods in annotating data from different species, platforms, and tissues, and also has reliable performance in the detection of unknown cell types. In addition, to test the robustness of scPML on cross-perturbation data, we conducted a cell type prediction experiment using the GSE96583 dataset (Supplementary Note 8, Supplementary Figs. 21 and 22).

From a technical perspective, scPML is a deep learning neural network model that has an advantage over correlation-based methods such as Seurat in identifying cell type patterns. Additionally, scPML simultaneously utilizes information on the relationships between cells and between genes in multiple pathways, and it can be seen that scPML can still learn cell type patterns well even in the presence of batch effects. We also demonstrated the benefits of combining information from multiple pathways. Moreover, we demonstrated that the inductive scPML model can efficiently perform cumulative learning on multiple training datasets using pre-training methods. In addition, we perform a sensitivity analysis on the primary hyperparameters of scPML and time complexity analysis, as detailed in Supplementary Note 2,  9 and Supplementary Figs. 13 and 23. Now, scPML is available for public use as a robust and reliable cell type prediction model.

Despite these successful results, there is still room for improvement in scPML. Firstly, as a neural network model, scPML has some limitations, such as model unexplainability, which can be addressed through downstream analysis such as differential gene identification and enrichment analysis, that can ameliorate some of the problems and bring insights into the labeled cells. Secondly, as a model with the ability to fuse multiple types of features, scPML should not be limited to constructing different cell-cell graphs using different pathways for complementary learning. Perhaps more diverse cell feature information, such as embedding from various other models, could also be considered. Notably, in Supplementary Note 5 and Supplementary Fig. 16, We integrate the cell-cell graph constructed using MNN (mutual nearest neighbors) with the gene features from the raw data, which has yielded improved performance. We have observed that using gene features at times produces better results than employing a single pathway alone. Therefore, for practical applications, the incorporation of gene features from the raw data as an additional view is recommended. We may explore this in future versions.

## Methods

### Construction of similarity matrix

In order to capture the topological characteristics of cells, we first need to calculate the similarities between single cells. The similarity between two cells can be represented by the euclidean distance between their features, which are often described by genes. A pathway is a collection of genes that regulates the same biological process^39^. Here, we use pathways to denote the features of single cells because pathway can better reduce the impact of dropout events as one gene has little impact on the entire gene set. We first partition single cells’ genes into many gene groups represented by pathways, then calculate the score for each cell-pathway pair. Specifically, we denote $X\in {{{{{{{{\mathcal{R}}}}}}}}}^{N\times M}$ as the gene expression data, where *N* is the number of cells in the training data and *M* is the number of genes. Then we need to utilize pathway data to calculate the pathway activity levels for each cell. We define the cell-pathway score matrix as $Q\in {{{{{{{{\mathcal{R}}}}}}}}}^{N\times {M}_{p}}$, where *M*_*p*_ is the number of pathways. The scoring process can be represented as:

1 $${Q}_{ij}={f}_{AUC}({X}_{i})$$

The mapping function *f*_*AUC*_ returns the Area Under Curve (AUC) where *x*-axis is the genes ranked by their counts decreasingly, *y*-axis is the number of genes that hits the pathway *j*. We use AUCell^33^ package in R to partition genes and calculate the cell-pathway scores.When calculating cellular pathway activity in this context, we do not need to consider other issues such as pathway topology information. Therefore, we choose simple and straightforward AUCell as score function. Once we get the cell-pathway score matrix, we can calculate the similarity between cells using Euclidean distance:

2 $${S}_{ij}=\frac{1}{1+\parallel {Q}_{i}-{Q}_{j}{\parallel }_{2}}$$

where $S\in {{{{{{{{\mathcal{R}}}}}}}}}^{N\times N}$ is the similarity matrix of single cell data and *S*_*ij*_ stands for the similarity between cell *i* and cell *j*.

The in-depth analysis of the advantages of pathways is provided in Supplementary Note 6,  7 and Supplementary Figs. 17–20.

### Graph construction

GCN^34^ takes as input the unweighted graph, which is often represented as an adjacent matrix. Here, based on similarity matrix, we use mutual nearest neighbors (MNN)^18^ concept to construct effective graph. First, we denote the adjacent matrix for $X\in {{{{{{{{\mathcal{R}}}}}}}}}^{N\times M}$ as $A\in {{{{{{{{\mathcal{R}}}}}}}}}^{N\times N}$. *A*_*ij*_ = 1 if cell *i* is the nearest neighbors of cell *j* and cell *j* is also the nearest neighbors of cell *i*, otherwise, *A*_*ij*_ = 0.

### Data pre-processing

For training and test data, we first take an intersection of their genes to obtain the common genes. We denote the training data as ${X}_{train}\in {{{{{{{{\mathcal{R}}}}}}}}}^{{N}_{0}\times {M}_{0}}$, and the test data as ${X}_{test}\in {{{{{{{{\mathcal{R}}}}}}}}}^{{N}_{1}\times {M}_{0}}$, where *N*_0_ and *N*_1_ are the number of cells in training data and test data, *M*_0_ is the number of shared high variable genes. As not all genes are useful for cell annotation. We use analysis of variance (ANOVA) to select top *M*_0_ = 2000 high variable genes (HVGs) across cell labels in training data. We keep only the HVGs in both training data and test data.

After gene feature selection, we performs median normalization for training data:

3 $${\hat{x}}_{ij}=\frac{{x}_{ij}}{\mathop{\sum }\nolimits_{j}^{{M}_{h}}{x}_{ij}}\frac{\mathop{\sum }\nolimits_{i}^{N0}\mathop{\sum }\nolimits_{j}^{{M}_{h}}{x}_{ij}}{{N}_{0}}$$

where *x*_*ij*_ is the raw value of cell *i* and feature *j* in *X*_*train*_ and ${\hat{x}}_{ij}$ is the normalized value, where 1 ≤ *i* ≤ *N*_0_ and 1 ≤ *j* ≤ *M*_*h*_. We make each cell have the same expression counts as the average across cells. By doing so we can eliminate the impact of cell size. After normalization, training data becomes ${\hat{X}}_{train}$. For the test data, we follow the similar fashion and normalize test data as ${\hat{X}}_{test}$.

### Obtaining low-dimensional representations through graph auto-encoder

To incorporate the structural information of single cells and reduce the noise, we design a self-supervised graph auto-encoder. For training data ${\hat{X}}_{train}$, We first randomly mask some non-zero values and attempts to reconstruct these values through the graph auto-encoder. The graph auto-encoder consists of an encoder and decoder both based on graph convolutional network^34^. The encoder takes as input the randomly masked expression matrix ${\hat{X}}_{train}$ and the according cell-cell graph *A*_*train*_. Then the encoder aggregate the hierarchical cell information to produce low-dimensional representation for each cell, which is denoised and incorporates the knowledge of high-order relations between cells. The decoder maps the low-dimensional representation to original feature space and attempts to reconstruct the masked values of raw data. Formally, with a learnable matrix ${W}^{(1)}\in {{{{{{{{\mathcal{R}}}}}}}}}^{M\times d}$ as the parameters of encoder, where *d* < < *M*, and a non-linear function *σ*, the encoder can be defined as:

4 $${H}_{train}=\sigma \left({\widetilde{A}}_{train}{\hat{X}}_{train}{W}^{(1)}\right)$$

where *H*_*train*_ is the low-dimensional output of encoder and we use *ReLu* as the non-linear function. For efficiency^34^, we normalize *A*_*train*_ as ${\widetilde{A}}_{train}={\tilde{D}}_{train}^{-\frac{1}{2}}({A}_{train}+I){\widetilde{D}}_{train}^{-\frac{1}{2}}$, where *I* is the identity matrix, ${\tilde{D}}_{train}$ is the diagonal degree matrix of (*A*_*train*_ + *I*).

The decoder performs the same propogation rules on H and produce the reconstructed matrix ${\widetilde{X}}_{train}\in {{{{{{{{\mathcal{R}}}}}}}}}^{{N}_{0}\times {M}_{0}}$:

5 $${\widetilde{X}}_{train}=\sigma ({\widetilde{A}}_{train}{H}_{train}{W}^{(2)})$$

where ${W}^{(2)}\in {{{{{{{{\mathcal{R}}}}}}}}}^{d\times {M}_{0}}$ is the parameter of decoder.

For the parameters optimization, we define the loss function of training as:

6 $$\min {{{{{{{{\mathcal{L}}}}}}}}}_{r}=\min {\left\Vert {\hat{X}}_{train\_m}-{\widetilde{X}}_{train\_m}\right\Vert }_{2}$$

where *X*_*train*_*m*_ and ${\widetilde{X}}_{train\_m}$ are the masked values we retrieve from ${\hat{X}}_{train}$ and ${\widetilde{X}}_{train}$ respectively.

For test data, we use the encoder with parameters estimated from training data to obtain low-dimensional representations, which can be represented as:

7 $${H}_{test}=\sigma \left({\widetilde{A}}_{test}{\hat{X}}_{test}{W}^{(1)}\right)$$

With various pathway datasets, we can construct multiple cell-cell graphs from different perspectives. Let ${\{{A}^{{v}_{i}}\}}_{i = 1}^{{N}_{v}}$ be the set of multiple cell-cell graphs, where *N*_*v*_ is the number of pathway datasets, i.e. the number of views. In our experiments, we utilize four distinct pathway datasets (namely, KEGG, Reactome, Wikipathway, and Yan) with scPML, thus *N*_*v*_ = 4.We use multiple independent graph auto-encoders to encode the data following the same fashion, then we can obtain the training representation set ${\{{H}_{train}^{{v}_{i}}\}}_{i = 1}^{{N}_{v}}$ and test representation set ${\{{H}_{test}^{{v}_{i}}\}}_{i = 1}^{{N}_{v}}$ respectively.

### Multi-view learning

In scPML, we can describe each cell from different views with multiple representations produced by the graph encoder, denoted as ${\{{H}^{{v}_{i}}\}}_{i = 1}^{{N}_{v}}$. In order to sufficiently utilize these views to obtain a complementary representation, we use multi-view learning to integrate them by using latent subspace learning method^44^. Intuitively, we want to find a common latent representation denoted as $h\in {{{{{{{{\mathcal{R}}}}}}}}}^{N\times {d}_{s}}$ (*N* is the number of cells and *d*_*s*_ is the dimension of features in latent subspace) that can reflects the characteristics of representations of different views. Following the previous study^44^, we define a set of mapping functions as ${f}_{{v}_{j}}({h}_{i};{\theta }_{{v}_{j}}),1\le i\le N,1\le j\le {N}_{v}$, which attempts to reconstruct the common representation *h*_*i*_ back to the original representations of different views. The reconstruction loss can be represented as:

8 $${\ell }_{r}(h,\theta )=\mathop{\sum }\limits_{i=1}^{N}\mathop{\sum }\limits_{j=1}^{{N}_{v}}{\left\Vert {f}_{{v}_{j}}({h}_{i};{\theta }_{{v}_{j}})-{H}_{i}^{({v}_{j})}\right\Vert }_{2}$$

where $${h}_{i}\in {{{{{{{{\mathcal{R}}}}}}}}}^{{d}_{s}},{H}_{i}\in {{{{{{{{\mathcal{R}}}}}}}}}^{d}$$.

In order to make the latent representation structured for separability^44^, we incorporate label information by adding misclassification loss:

9 $$\begin{array}{rcl}{\ell }_{c}({y}_{i},y,{h}_{i})&=&\mathop{\sum }\limits_{i=1}^{N}\mathop{\max }\limits_{y\in {{{{{{{\mathcal{Y}}}}}}}}}\left.\right(0,{{\Delta }}({y}_{i},y)\\ &&+{{\mathbb{E}}}_{h \sim \tau (y)}F(h,{h}_{i})-{{\mathbb{E}}}_{h \sim {{{{{{{{\mathcal{T}}}}}}}}}_{{y}_{i}}}F(h,{h}_{i})\left.\right)\end{array}$$

where $F(h,{h}_{i})={h}^{T}{h}_{i},{{{{{{{\mathcal{Y}}}}}}}}$ is the set of class labels. *τ*(*y*) is the set of latent representation with class *y*. Δ(*y*_*n*_, *y*) = 0 if *y* = *y*_*n*_, else Δ(*y*_*n*_, *y*) = 1. Intuitively, we can see that the misclassification loss attempts to maximize ${{\mathbb{E}}}_{h \sim \tau (y)}F(h,{h}_{n})$ and minimize ${{\mathbb{E}}}_{h \sim \tau ({y}_{n})}F(h,{h}_{n})$, which will make the similarity between *h* with the same class *y*_*i*_ larger than that *h* with different labels by a margin Δ(*y*_*n*_, *y*).

The overall objective loss function of multi-view learning is deduced as:

10 $$\mathop{\min }\limits_{({\{{h}_{i}\}}_{i = 1}^{N},{\{{\theta }_{{v}_{i}}\}}_{i = 1}^{{N}_{v}})}{{{{{{{{\mathcal{L}}}}}}}}}_{m}=\frac{1}{N}({\ell }_{r}+\lambda {\ell }_{c})$$

where *λ* > 0 balances the weight of information from multiple views and class labels.

At training stage, we randomly initialize the parameters ${\{{\theta }_{{v}_{i}}\}}_{i = 1}^{{N}_{v}}$ of the mapping ${\{{f}_{{v}_{i}}\}}_{i = 1}^{{N}_{v}}$ and the common latent representations *h*. Then the ${\{{\theta }_{{v}_{i}}\}}_{i = 1}^{{N}_{v}}$ and *h* are optimized by minimizing reconstruction loss *ℓ*_*r*_ and ${{{{{{{{\mathcal{L}}}}}}}}}_{m}$ respectively by using stochastic gradient descent:

11 $${\theta }_{{v}_{i}}\leftarrow {\theta }_{{v}_{i}}-\frac{1}{N}\alpha \frac{\partial {\ell }_{r}}{\partial {\theta }_{{v}_{i}}}$$

12 $${h}_{trai{n}_{i}}\leftarrow {h}_{trai{n}_{i}}-\alpha \frac{1}{{N}_{0}}\frac{\partial {{{{{{{{\mathcal{L}}}}}}}}}_{m}}{\partial {h}_{trai{n}_{i}}}$$

where *α* is the learning rate. The optimization will stop if the misclassification loss becomes convergent or the iterations exceeds the maximum epochs we set.

At testing stage, we preserve the parameters ${\{{\theta }_{{v}_{i}}\}}_{i = 1}^{{N}_{v}}$ estimated from training process and calculate the latent representations for test data using stochastic gradient descent:

13 $${h}_{tes{t}_{i}}\leftarrow {h}_{tes{t}_{i}}-\frac{1}{{N}_{1}}\alpha \frac{\partial {\ell }_{r}}{\partial {h}_{tes{t}_{i}}}$$

The pseudocode for the training and test procedure of multi-view learning can be summarized as Table 1.

**Table 1:** The training and test procedure of multi-view learning.

Training stage
Input: representation of training data ${\{{H}_{train}^{{v}_{i}}\}}_{i = 1}^{{N}_{v}}$ and labels ${\{{y}_{i}\}}_{i = 1}^{N}$
Initialization: Randomly initialize the values of ${\{{\theta }_{{v}_{i}}\}}_{i = 1}^{{N}_{v}}$ and ${h}_{train}\in {{{{{{{{\mathcal{R}}}}}}}}}^{{N}_{0}\times {d}_{s}}$
For *epoch* → *epochs*
For *j* = 1 → *N*_*v*_
Optimize the parameters of mapping functions: ${\{{f}_{{v}_{j}}\}}_{j = 1}^{{N}_{v}}$
$${\theta }_{{v}_{j}}\leftarrow {\theta }_{{v}_{j}}-\frac{1}{{N}_{0}}\alpha \frac{\partial {\ell }_{r}}{\partial {\theta }_{{v}_{j}}}$$
End For
For *i* = 1 → *N*_0_
Optimize the latent representation *h*_*train*_ for training data:
$${h}_{trai{n}_{i}}\leftarrow {h}_{trai{n}_{i}}-\frac{1}{{N}_{0}}\alpha \frac{\partial {{{{{{{{\mathcal{L}}}}}}}}}_{m}}{\partial {h}_{trai{n}_{i}}}$$
End For
End For
Output: the parameters ${\{{\theta }_{{v}_{i}}\}}_{i = 1}^{{N}_{v}}$ and latent representation of training data *h*_*train*_
Test Stage
Use the parameters of mappings functions estimated from training data to update latent representation of test data
For *epoch* → *epochs*_*test*_
For *i* → *N*_1_
$${h}_{tes{t}_{i}}\leftarrow {h}_{tes{t}_{i}}-\frac{1}{{N}_{1}}\alpha \frac{\partial {\ell }_{r}}{\partial {h}_{tes{t}_{i}}}$$
End For
For

### Classification module

We use a two-layer fully connected neural network as the classification module. Formally, we define *F*_*i*_ as the *i* − *th* fully connected layer. The forward propagation is realized as:

14 $${\hat{y}}_{n}=softmax\left.\right({F}_{2}(ReLU({F}_{1}({h}_{n})))$$

where the softmax function is represented as:

15 $$softmax(x)=\frac{exp(x)}{\sum exp(x)\left.\right)}$$

Subsequently, We optimize the classification module using the following cross-entropy loss:

16 $${{{{{{{{\mathcal{L}}}}}}}}}_{CE}=-\frac{1}{N}\mathop{\sum }\limits_{i=1}^{N}{y}_{i}log({\hat{y}}_{i})$$

Furthermore, we conduct a performance comparison using different classification methods, as detailed in Supplementary Note 3 Supplementary Fig. 14.

### Cross-species classification

For cross-species classification, we select homologous genes between human and mouse and keep only genes that have a one-to-one correspondence by using HomoloGene databases. We keep the homologous genes that overlap between the mouse and human data and we convert mouse gene names to human gene names to obtain compatible input for graph construction with pathways.

### Multiple-training data

With multiple training data, we first take an intersection of their gene features and for the sake of simplicity, we only keep the HVGs selected from the first training data as their gene features. Given a training sequence like (training data 1, training data 2, …), we will first train scPML model with training data 1 and save the model. Then we load the model trained with training data 1 and feed it with training data 2, and so on. After finishing training, we will use the final model to predict cells in test data.

### Comparison methods

We compared our model with other methods including Seurat^19^, scmap^15^, SingleR^16^, CHETAH^17^, scGCN^24^, scArches^37^ and Geneformer^36^. For Seurat, we use Seurat V4 with default Principle Component Analysis (PCA) as reduction method. For scmap, We annotate cell types of test data using the scmapCluster function. For CHETAH, we predict cell types in test data using the CHETAHclassifier function. For SingleR, we use the SingleR function. For Geneformer, we use 6-layer pre-trained model. For scArches, we use treeArches^38^ model to annotate cells. All the methods are applied with default parameters. For more information see Supplementary Table 2.

### Statistics and reproducibility

All data are publicly available online and detaila information (e.g. sample size) can be seen in Supplementary Table 1. All experiments can be reproduced by using the code and hyperparameters we provide (See code availability).

### Reporting summary

Further information on research design is available in the Nature Portfolio Reporting Summary linked to this article.

### Supplementary information

Supplementary Information

Description of Additional Supplementary Files

Supplementary Data

Reporting Summary

## Data Availability

All datasets analyzed in the current study are publicly available and can be downloaded from their public accessions. The PBMC data of six different sequencing protocols are available from the Broad Institute Single Cell portal (https://portals.broadinstitute.org/single_cell/study/SCP424/single-cell-comparisonpbmc-data)^47^. The published pancreatic datasets were downloaded from https://hemberg-lab.github.io/scRNA.seq.datasets/(Baron^52^, Xin^53^, Muraro^54^, Segerstolpe^55^). The source data of mouse liver were downloaded from https://bis.zju.edu.cn/MCA/^57^. The source data of tumor were downloaded from https://www.ncbi.nlm.nih.gov/geo/(GSE72056, GSE10332, GSE118056, GSE117988)^58,59^. The source data of Cao^60^ were downloaded https://cblast.gao-lab.org/download. The source data of cross-perturbation were downloaded from GSE96583. The pathway datasets used in this paper can be downloaded from https://github.com/GaoLabXDU/sciPath. For detailed data information see Supplementary Table 1 and Supplementary Note 1.

## Code availability

The source code of scPML are implemented as a python software that is freely available at Github^61^ (https://github.com/Kevis9/Cell_Classification). The source code for comparison methods are publicly available. (See Supplementary Table 2).
