# Supplementary material for: scPML: pathway-based multi-view learning for cell type annotation from single-cell RNA-seq data
Source: Commun Biol. 2023 Dec 14;6:1268. doi: 10.1038/s42003-023-05634-z (PMC10721875; doi:10.1038/s42003-023-05634-z)
Supplement: Supplementary file 1 — Supplementary Information [file 42003_2023_5634_MOESM1_ESM.pdf]

SUPPLEMENTARY INFORMATION

scPML:Pathway-based Multi-view Learning for Cell Type Annotation from Single-cell RNA-seq Data

Supplementary Table 1. Datasets analyzed in this paper

Dataset	Organism	Tissue	Number of cells	Experiment protocol	Cell Population Number
PBMCs[1]	Human	PBMC	526	CEL-Seq2	7
			3222	10x Chromium (v3)	8
			6584	Drop-seq	9
			6584	inDrop	9
			3773	Seq-Well	8
			526	Smart-seq2	7
Baron[2]	Mouse	Pancreas	1886	InDrop	13
	Human	Pancreas	8569	InDrop	14
Xin[3]	Pancreas	Pancreas	1449	SMARTer	4
Segerstolpe[4]	Pancreas	Pancreas	2133	SMART-Seq2	13
Muraro[5]	Pancreas	Pancreas	2122	CEL-Seq2	9
GSE72056[6]	Human	Tumor	3280	SMART-Seq2	7
GSE10332	Human	Tumor	4570	SMART-Seq2	5
GSE118056[7]	Human	Tumor	6043	10X	8
GSE117988[7]	Human	Tumor	10082	10X	5
Cao_2020_kidney[18]	Human	Kidney	155386	sci-RNA-seq3	9

Supplementary Table 2. Methods compared in this paper

Method	Software	Unknown cell types allowed	Algorithm	Version	Source
scGCN[8]	Python	Yes	Graph convolutional network		https://github.com/QSong-github/scGCN/
Seurat[9]	R	No	Anchor discovery based on CCA and MNN, and weighted vote classifier based distance	4.0.3	https://satijalab.org/seurat/articles/install.html
SingleR[10]	R	No	Correlation-based with Spearman	1.6.1	https://bioconductor.org/packages/release/bioc/html/SingleR.html
CHETAH[11]	R	Yes	Correlation-based with hierarchical classification	1.0.0	https://www.bioconductor.org/packages/release/bioc/html/CHETAH.html
scmap-cluster[12]	R	No	Correlation-based with Cosine, Spearman, Pearson	1.14.0	https://bioconductor.org/packages/release/bioc/html/scmap.html
Geneformer	Python	Yes	Transformer		https://huggingface.co/ctheodoris/Geneformer
scArches	Python	No	Architecture surgery to project query on the top of reference datasets and applies to different models.		https://docs.scarches.org/en/latest/about.html

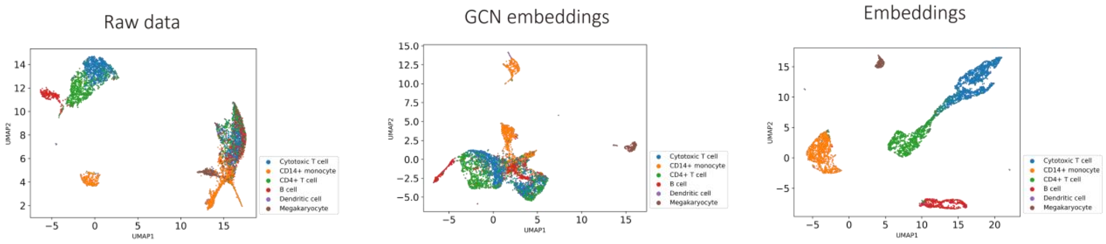

Supplementary Figure 1. UMAP projections of scPML for SeqWell-10X_V3 experiments. First column is the raw data. Second column is the embedding obtained from self-supervised GCN. Third column is the embedding obtained from classifier. Cells are marked with true labels.

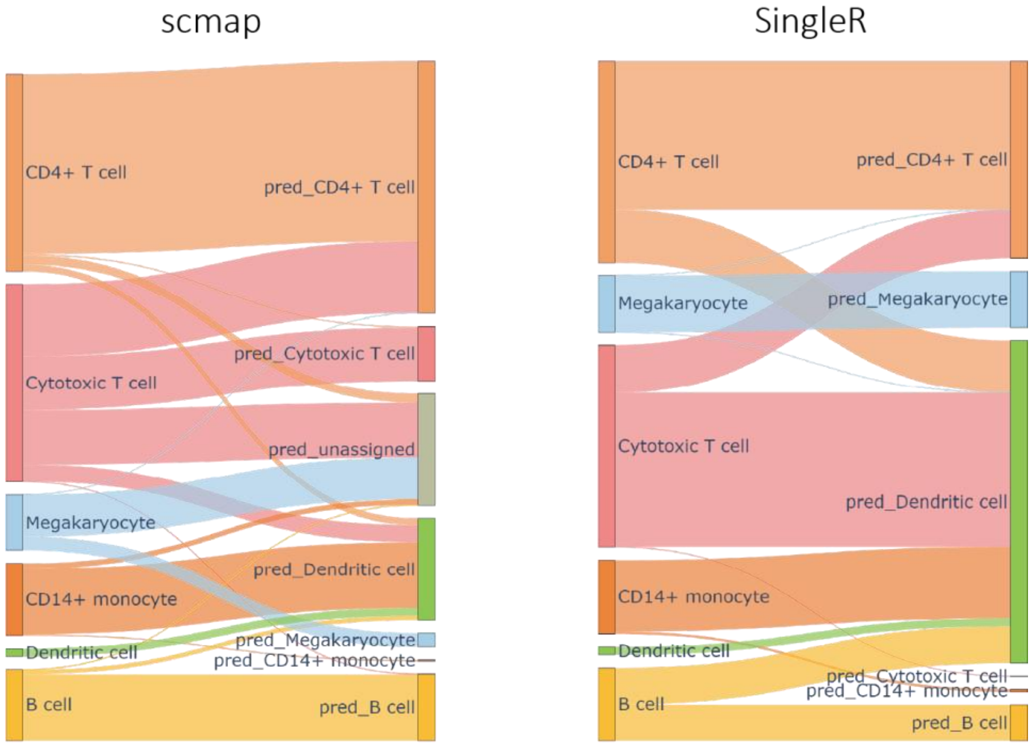

Supplementary Figure 2. Sankey plots of scmap and SingleR for SeqWell_10X_V3. Left column represents the true labels. Right column represents the labels predicted by scPML.

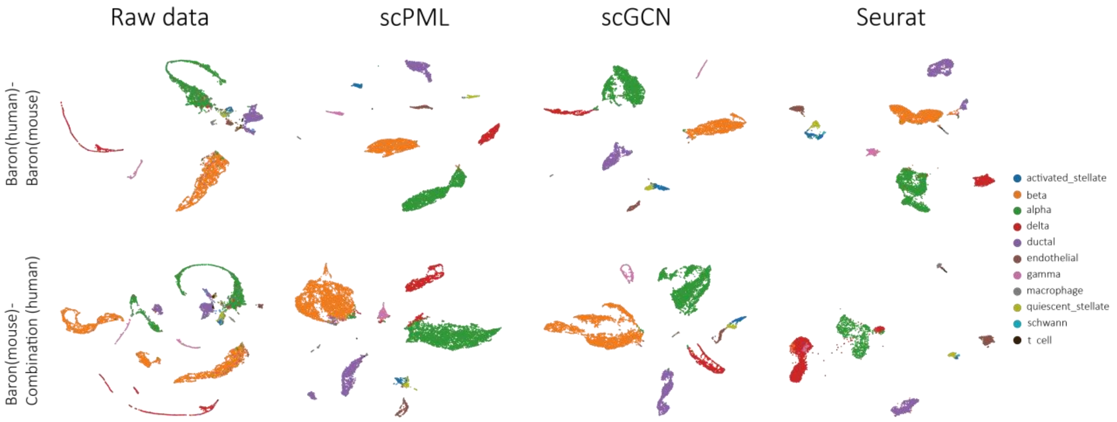

Supplementary Figure 3. UMAP projections for Baron(mouse)-Baron(human) and Baron(mouse)-Combination(human) for raw data, scPML, scGCN and Seurat with cells marked by predicted labels.

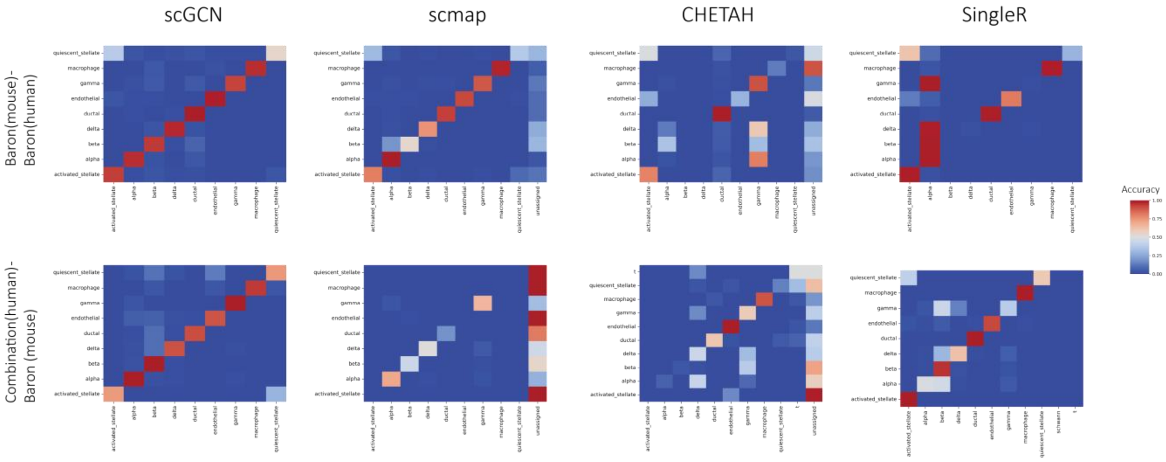

Supplementary Figure 4. Confusion matrix for Baron(mouse)-Baron(human) and Combination(human)-Baron(mouse) of scGCN, scmap,CHETAH and SingleR.

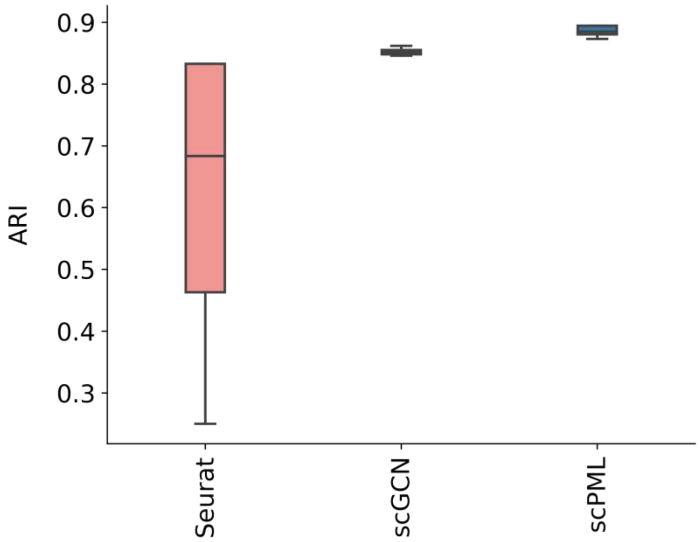

Supplementary Figure 5. Box plot of ARI of Seurat, scGCN and scPML for cross-species experiments. The middle line is the median. The lower and upper hinges represents the first and third quartiles, with the whiskers in the range of 1.5-times the inter-quartile(IQR)

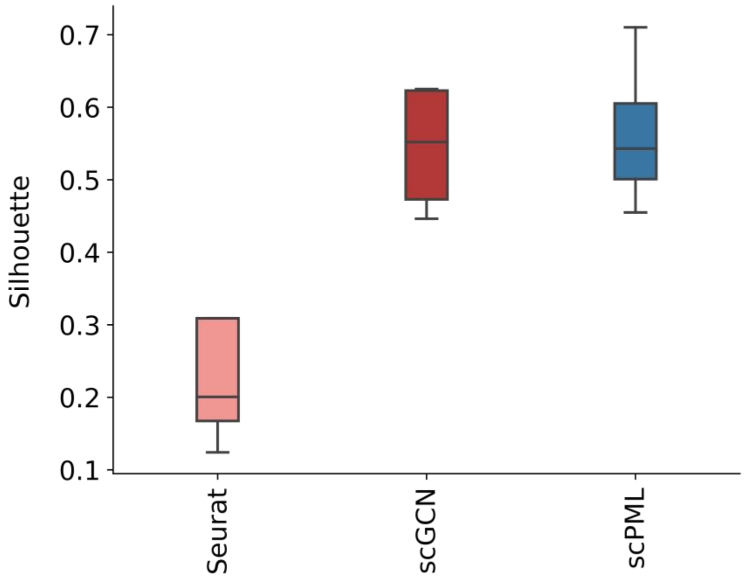

Supplementary Figure 6. Box plot of Silhouette score of Seurat, scGCN and scPML for cross-species experiments. In the box plot, the middle line represents the median, the lower and upper hinges represent the first and third quartiles, and the whiskers extend to the range of 1.5 times the interquartile range (IQR).

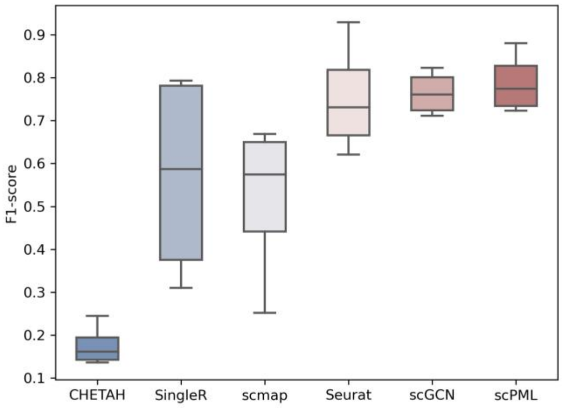

Supplementary Figure 7. The Macro F1 of cross-species experiments for all cell annotation methods. In the box plot, the middle line represents the median, the lower and upper hinges represent the first and third quartiles, and the whiskers extend to the range of 1.5 times the interquartile range (IQR). The average Macro F1 score of scPML is 0.79. scGCN: 0.76. Seurat: 0.75. SingleR: 0.57. scmap: 0.52. CHETAH: 0.18.

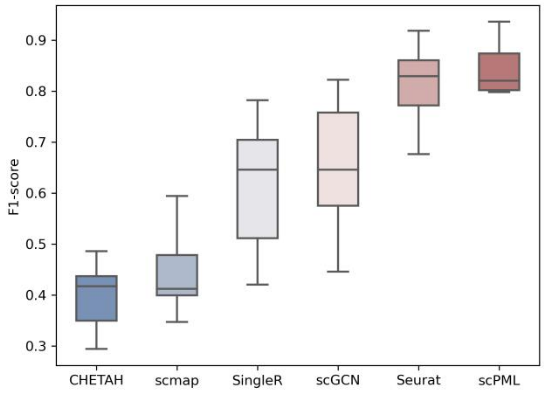

Supplementary Figure 8. The Macro F1 of cross-platform experiments for all cell annotation methods. In the box plot, the middle line represents the median, the lower and upper hinges represent the first and third quartiles, and the whiskers extend to the range of 1.5 times the interquartile range (IQR). The average Macro F1 score of scPML is 0.82. Seurat: 0.80. scGCN: 0.65. SingleR: 0.61. scmap: 0.45. CHETAH: 0.42.

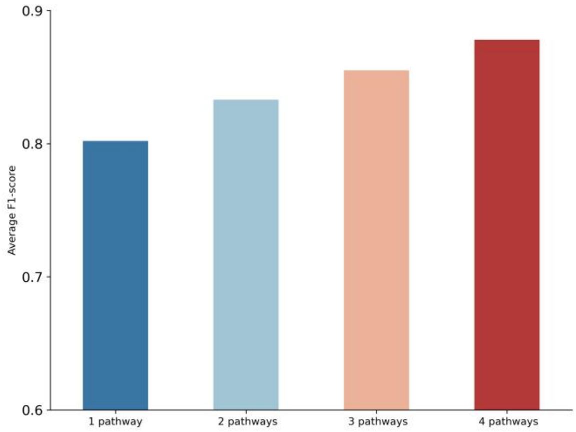

Supplementary Figure 9. The average Macro F1 of the benefits of multi-view learning experiments. 1 pathway, 2 pathways, 3 pathways, and 4 pathways refer to the number of pathways used in the experiments. Throughout the entire study, we utilized KEGG, Reactome, and Wikipathway and Yan these four pathway datasets.

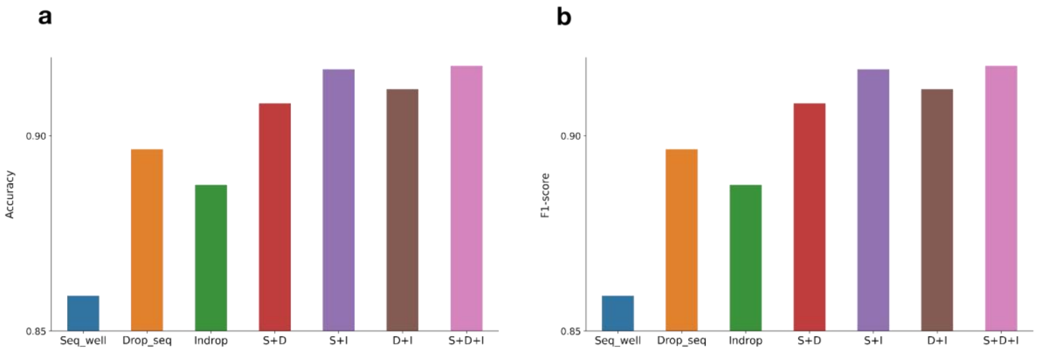

Supplementary Figure 10. Multiple training data experiments on PBMCs dataset ,including Seq_well, Drop_seq and Indrop these three datasets. We use initial letter representing different datasets. **a,** The accuracy on different experiments .**b**,The Macro F1 of different experiments.

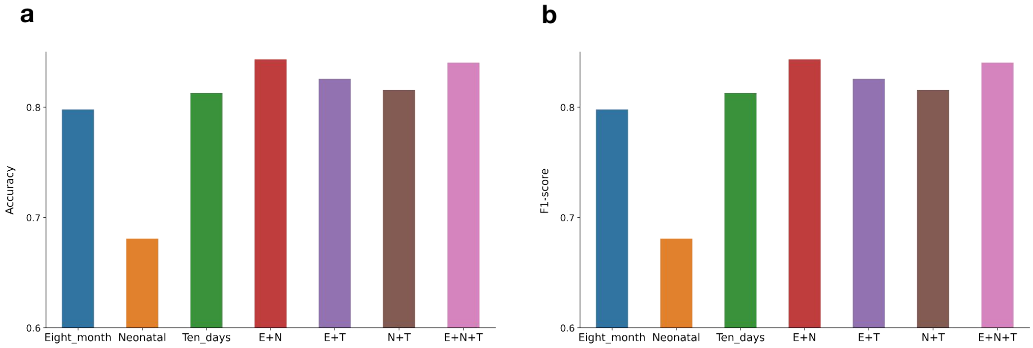

Supplementary Figure 11. Multiple training data experiments on MCA_liver dataset. We use initial letter representing different datasets. **a**, The accuracy on different experiments. **b**,The Macro F1 of different experiments.

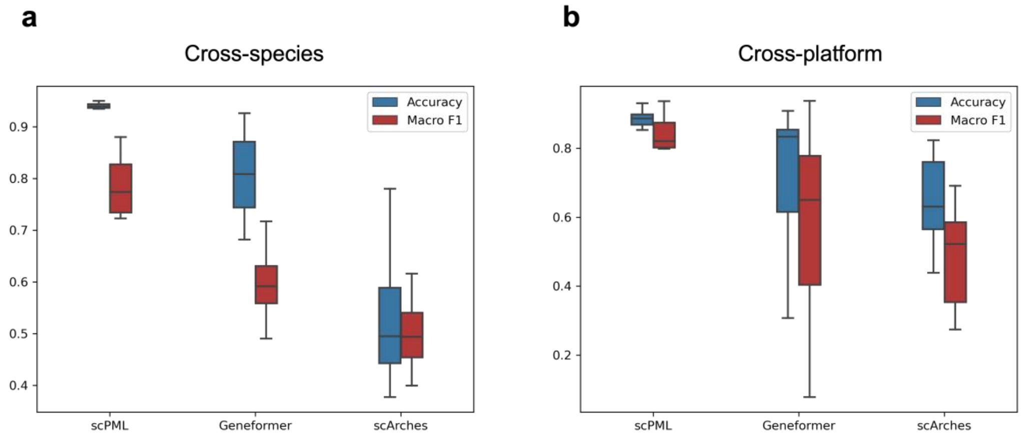

Supplementary Figure 12. Comparison of scPML with Geneformer and scArches in cross-species and cross-platform experiments. **a**, Box plots are used to illustrate the accuracy and Macro F1 of all methods in cross-species experiments. The blue bar shows accuracy score and the red bar shows Macro F1 score. In the box plot, the middle line represents the median, the lower and upper hinges represent the first and third quartiles, and the whiskers extend to the range of 1.5 times the interquartile range (IQR). scPML achieved the best performance with an average accuracy and Macro F1 score of 0.94 and 0.79, higher than Geneformer (mean acc=0.81, mean macro f1 = 0.60) and scArches (mean acc=0.54, mean macro f1=0.50). **b**, Accuracy and Macro F1 score of scPML, Geneformer and scArches in cross-platform experiments. In the box plot, the middle line represents the median, the lower and upper hinges represent the first and third quartiles, and the whiskers extend to the range of 1.5 times the interquartile range (IQR). For cross-platform experiments, scPML also achieved the best performance with an average accuracy and Macro F1 score of 0.87 and 0.82, higher than Geneformer (mean acc=0.72, mean macro f1=0.58) and scArches (mean acc=0.65, mean macro f1=0.48).

Supplementary Note 1: Pathway datasets used in this paper

In this paper, we used four pathway datasets to construct various cell-cell graphs. The details of four pathway datasets are as follows:

KEGG[13]: Knowns as Kyoto Encyclopedia of Genes and Genomes. The creation of KEGG involved the manual compilation of information on regulatory and metabolic connections among genes, proteins, and compounds. In this paper, we included 396 pathways of humans.

Reactome[14]: Our study utilized version 3.1 of Reactome, a pathway database that has been meticulously curated and reviewed by experts, and contains a vast amount of information on gene regulation. Specifically, we analyzed 2213 pathways in the human system.

WikiPathway[15]: The ConsensusPathDB (CPDB) database provided us with access to 601 human pathways that we downloaded from the multifaceted Wikipathways pathway database for our study.

De no va pathway[16]: Ji et al. presented a novel approach for creating functional pathways by performing hierarchical gene clustering on each scRNA-seq dataset, which resulted in the default generation of 150 de novo pathways.

We obtained these pathway data in the gmt file format from Zhang[17].

Supplementary Note 2: Sensitivity analysis on main hyper-parameters

In order to assess how hyper-parameter configurations affect the accuracy of predictions, we conducted cross-species and cross-platform experiments, which totally involved 15 training-testing data pairs. Supplementary Figure 8 shows the line plot of accuracy of different experiments with different hyper-parameter settings. We can see that the performance keeps steady for most hyper parameters. For some data pairs, k_neighbor parameter plays an important role. According to these results, we can have a clear outline for main hyper-parameter settings. For instance, a suitable value for the hyper-parameter k_neighbor would be approximately 5.

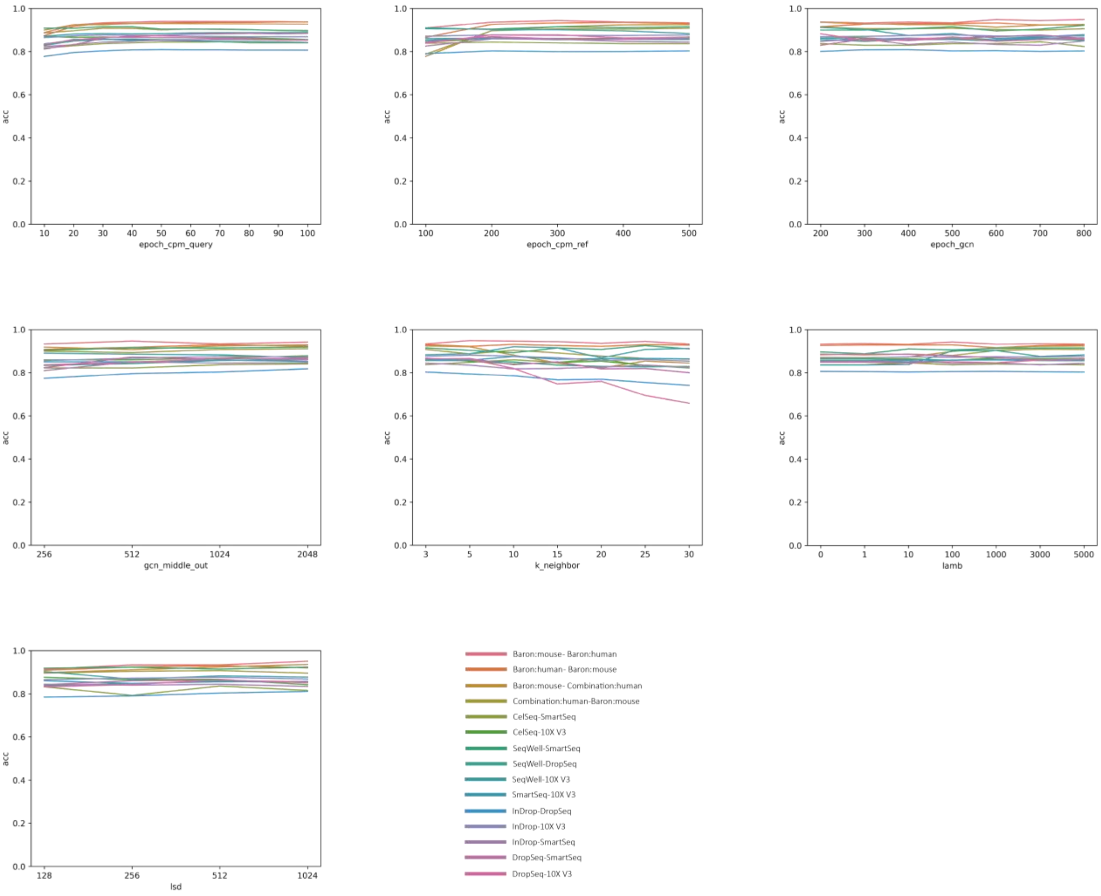

Supplementary Figure 13. Accuracy of cross-species and cross-platform experiments with different hype-parameter settings.

Supplementary Note 3: Performance with different classification methods

With latent representation obtained from multi-view learning module, we can use many methods to classify them. A simple method is to plug in fully connected layers network to learn the patterns of training latent representations and use the trained network to classify test latent representation. Another way is to calculate the similarity between test representations and training representations and choose the the most similar training cell's label as predicted label:

$$y_{test_i} = \arg\max_{y \in \mathcal{Y}} \mathbb{E}_{\mathbf{h} \sim \mathcal{T}(y)} F\left(\mathbf{h}, \mathbf{h}_{test_i}\right)$$

Here, we compared the performance of these two methods with accuracy score on cross-species and cross-platform experiments(Supplementary Figure 9). We can see that classifier made up of fully connected layers achieved a better performance on cross-species and cross-platform experiments than similarity calculation methods.

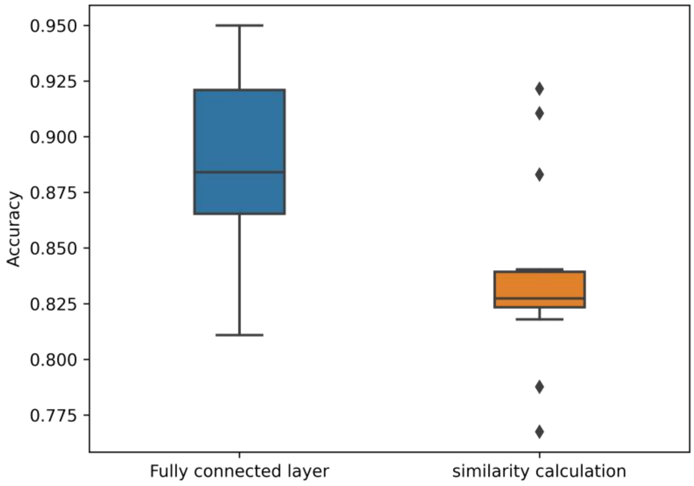

Supplementary Figure 14. Box plot of the performance of two different classification methods based on latent representation obtained from multi-view learning. In the box plot, the middle line represents the median, the lower and upper hinges represent the first and third quartiles, and the whiskers extend to the range of 1.5 times the interquartile range (IQR).

Supplementary Note 4: Performance of reversed cross-platform experiments

The cross-platform experiments involves 11 experiments: CelSeq-SmartSeq, CelSeq-10X V3, SeqWell-SmartSeq, SeqWell- DropSeq, SeqWell-10X V3, SmartSeq-10X V3, Indrop-DropSeq, Indrop-10X V3, Indrop-SmartSeq, DropSeq-SmartSeq, DropSeq-10X V3. In order to demonstrate the performance of scPML is independent of the selection of data pairs, we used the reversed training-test data pairs: SmartSeq-CelSeq, 10X V3-CelSeq, SmartSeq-SeqWell, DropSeq-SeqWell, 10X V3-SeqWell, 10X V3-SmartSeq, DropSeq-Indrop, 10X V3-Indrop, SmartSeq-Indrop, SmartSeq-DropSeq, 10X V3-DropSeq. As shown in Supplementary Figure 11, on average, scPML achieved the highest performance.

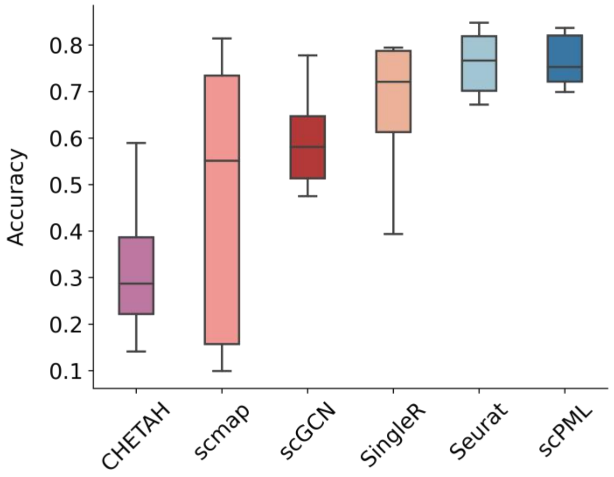

Supplementary Figure 15. Box plot of accuracy of all methods on reversed cross-platform data pairs. In the box plot, the middle line represents the median, the lower and upper hinges represent the first and third quartiles, and the whiskers extend to the range of 1.5 times the interquartile range (IQR).

Supplementary Note 5: Integrating additional view

Apart from views constructed using various pathway datasets, we have numerous options. In this study, we investigated whether utilizing a cell-cell graph constructed using gene features of cells as an additional view would enhance performance. We constructed the additional view as follows:

1. Selecting 2000 highly variable genes.
2. Using Principle Component Analysis(PCA) to reduce the dimension of feature.
3. Using Mutual Nearest Neighbors(MNN) to construct cell-cell graph.
4. Using self-supervised Graph Convolutional Network(GCN) to obtain middle embeddings.

As shown in Supplementary Figure 11, integrating view constructed from gene features improved the performance of classification.

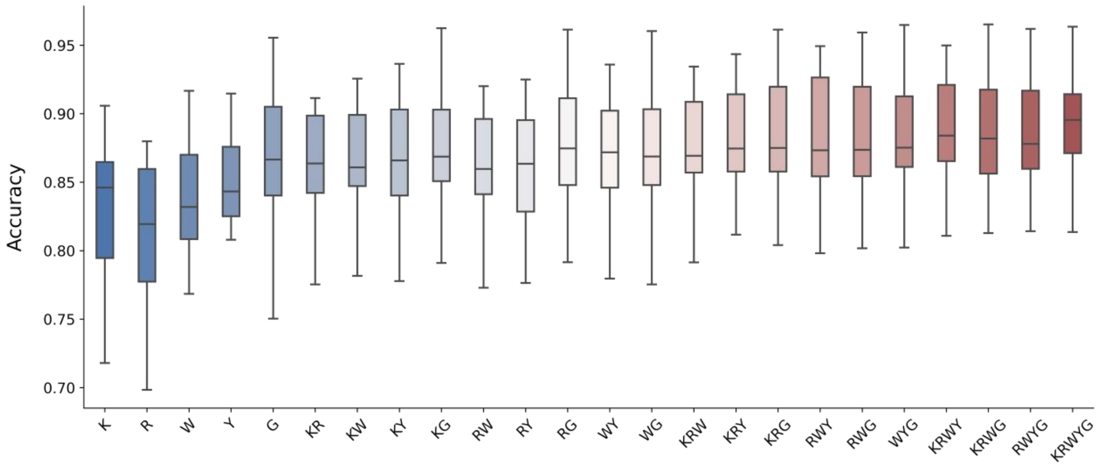

Supplementary Figure 16. Accuracy of cross-platform and cross-species using different view combinations. K stands for KEGG. R stands for Reactome. W stands for Wikipathway. Y stands for the yan pathway of de no va. G stands for view constructed only using gene features. In the box plot, the middle line represents the median, the lower and upper hinges represent the first and third quartiles, and the whiskers extend to the range of 1.5 times the interquartile range (IQR).

Supplementary Note 6: Ablation experiments of integrating pathways

We further investigated the contributions of pathways to scPML classification. Initially, a pathway ablation experiment was conducted, comparing the classification performance between integrating four pathways and using raw data for graph construction. The K-nearest neighbors (KNN) algorithm was employed for cell-cell graph construction using the original data, which is processed with the selection of 2000 high-variable genes. As depicted in Supplementary Figure 12, integrating four pathways exhibited higher accuracy and Macro F1 scores in classification compared to KNN. When constructing cell-cell graphs using pathways in cross-species data, the average classification accuracy was 94.1%, with a Macro F1 score of 79.0%, while KNN achieved an average accuracy of 84.0% and an F1 score of 64.5%. In the case of cross-platform data, the average accuracy for pathway-based classification was 87.1%, with an F1 score of 81.7%, while KNN yielded an average accuracy of 74.6% and a Macro F1 score of 68.8%. These results underscore the advantages of incorporating pathway-based graph information over using raw data for cell classification.

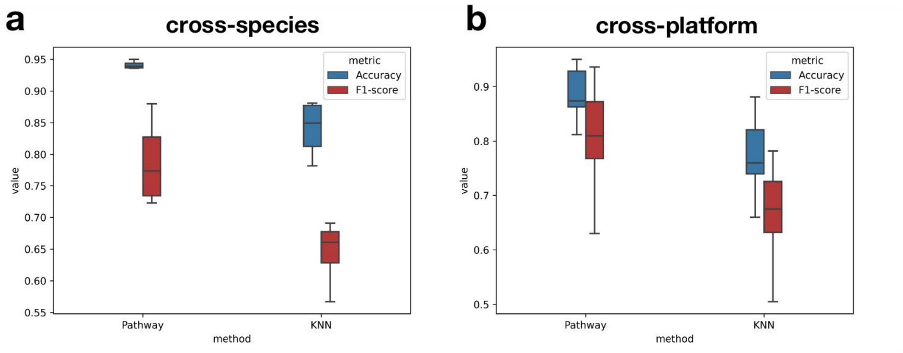

Supplementary Figure 17. The comparison of scPML on accuracy and Macro F1 scores between the integrating pathway and K-nearest neighbors (KNN) methods. **a**, Accuracy and Macro F1 scores for both methods in the cross-species experiment. **b,** Accuracy and Macro F1 scores for both methods in the cross-species experiment. In the box plot, the middle line represents the median, the lower and upper hinges represent the first and third quartiles, and the whiskers extend to the range of 1.5 times the interquartile range (IQR).

Supplementary Note 7: In-depth analyses of pathways

To visually illustrate the differences between constructing cell graphs with pathways and directly using raw data for graph construction, we employed heatmaps to visualize the adjacency matrices of four different pathways and the use of K-nearest neighbors (KNN). As shown in Supplementary Figure 13, darker color in the heatmap represents a higher number of connected cells between cell types. Firstly, it is evident that the four pathways exhibit differences in graph construction. For instance, KEGG shows a higher number of connections in B cells and CD14+ monocytes, while Reactome has fewer connections in B cells, and Wikipathway has fewer connections in dendritic cells. However, Wikipathway has more connections in CD4+ T cells and cytotoxic T cells compared to KEGG and Reactome, indicating unique advantages for different pathways. Secondly, we can observe that in cell types such as B cells, CD14+ monocytes, dendritic cells, and megakaryocytes, the four pathways provide more accurate cell connections than KNN. However, KNN performs better in distinguishing between CD4+ T cells and cytotoxic T cells. Therefore, we recommend considering the cell-cell graphs constructed from gene features as additional view to be integrated in practical usage.

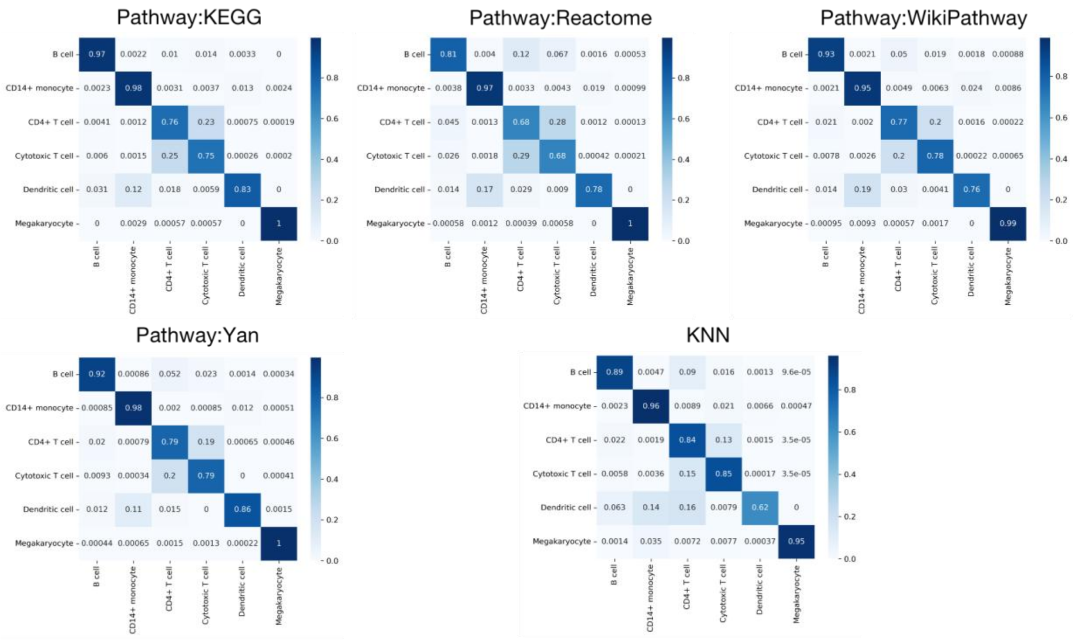

Supplementary Figure 18. Heatmaps of cell type connectivity counts for the four pathways and KNN on the 10X_V3 single-cell dataset. Darker color indicates a higher number of edges between cell types.

To further investigate the impact of gene information within pathways on classification, we performed random non-repetitive sampling on the original pathways at 10%, 30%, and 50% levels. We then studied their gene coverage, which refers to the proportion of genes present in the pathway compared to the genes in the original dataset, and its effect on the final scPML classification performance. We conducted these experiments for each pathway in both cross-species and cross-platform settings.

As shown in Supplementary Figure 13 and Supplementary Figure 14, we can clearly observe that for each individual pathway, as the pathway's information is reduced, scPML experiences a noticeable decrease in both accuracy and Macro F1 in classification. This may be attributed to the fact that higher gene coverage within pathways allows scPML to incorporate more informative data during graph construction, resulting in improved classification performance. Therefore, we recommend selecting pathway datasets with a higher amount of gene information when practical applications are considered.

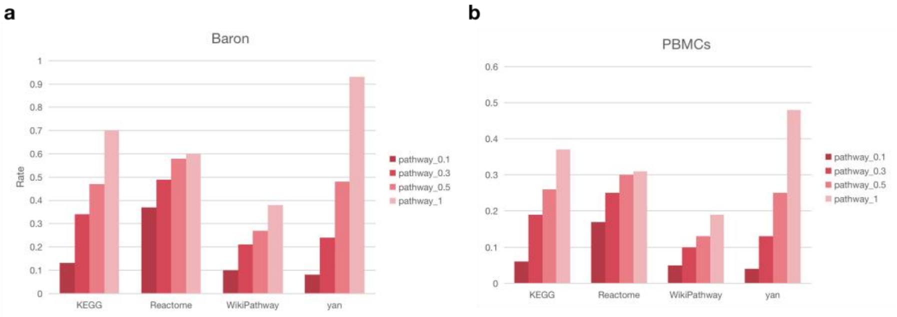

Supplementary Figure 19. The gene coverage rates of the KEGG, Reactome, WikiPathway, and Yan pathway datasets after random sampling at 10%, 30%, 50%, and under normal conditions. a, The gene coverage of the four pathways after different sampling proportions in the Baron dataset. b, The gene coverage of the four pathways after different sampling proportions in the PBMCs dataset.

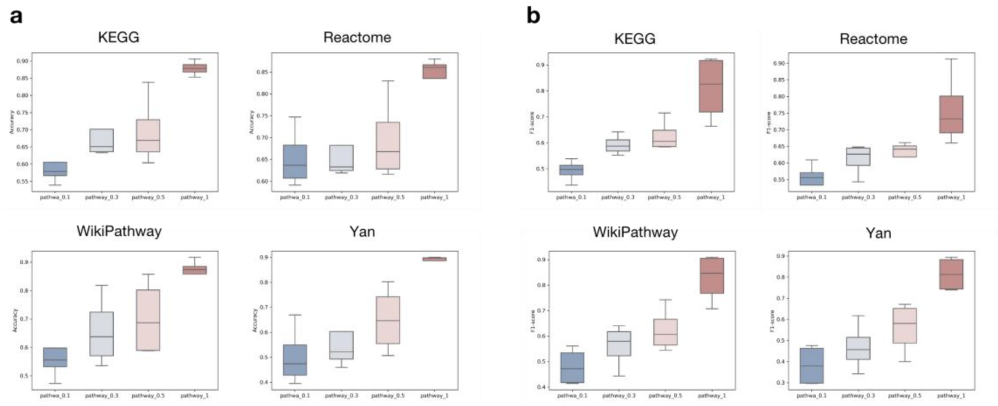

Supplementary Figure 20. The performance of four individual pathways under different levels of gene information. We use box plots to show the results, where the middle line represents the median, the lower and upper hinges represent the first and third quartiles, and the whiskers extend to the range of 1.5 times the interquartile range (IQR). **a**, The accuracy of the four distinct pathways when randomly sampled at proportions of 0.1, 0.3, 0.5, and with complete pathway information. 'Pathway_0.1' represents a random sampling of 10% of pathway information, 'Pathway_0.2' represents 20%, 'Pathway_0.5' represents 50%, and 'Pathway_1' represents the original pathway data. **b**, The Macro F1 scores of the four distinct pathways when randomly sampled at proportions of 0.1, 0.3, 0.5, and with complete pathway information.

Supplementary Note 8: Cross-perturbation experiments

To test the robustness of scPML on cross-perturbation data, we conducted a cell type prediction experiment using the GSE96583 dataset. GSE96583 comprises samples subjected to six-hour IFN-beta stimulation (Sample A) and unstimulated samples (Sample B). Due to the stimulation with IFN-beta, immune responses and antiviral mechanisms in cells from Sample A are activated, potentially altering the gene expression patterns and interfering with cell type-specific gene features. In this context, we employed Sample B as the training data and Sample A as the test data. The results showed that scPML, Seurat, scGCN, CHETAH, scmap, and SingleR all exhibited suboptimal cell annotation performance on cross-perturbation data, as depicted in Supplementary Figure 15. These methods struggled to cope with the interference. However, scPML still provided good performance in cell clustering, as shown in Supplementary Figure 16. We presented UMAP projections of the original data and scPML's cell clustering and provided quantitative evaluation metrics, including Macro F1 and Silhouette scores. The distribution of raw data was influenced by batch effects. The Silhouette score and batch cross-entropy for raw data were -0.118 and -2931.59, respectively. For scPML, the Silhouette score and batch cross-entropy were 0.538 and -2895.94, respectively, indicating that scPML alleviated the batch effects between samples.

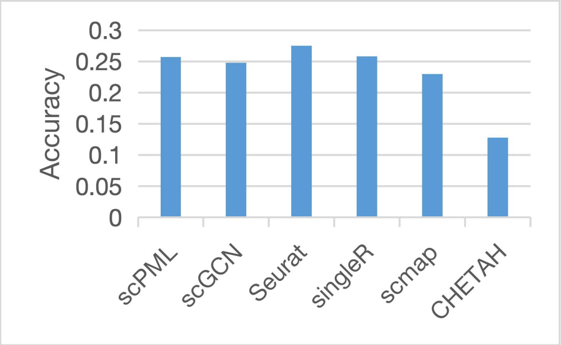

Supplementary Figure 21. Accuracy of all methods for cross-perturbation experiments.

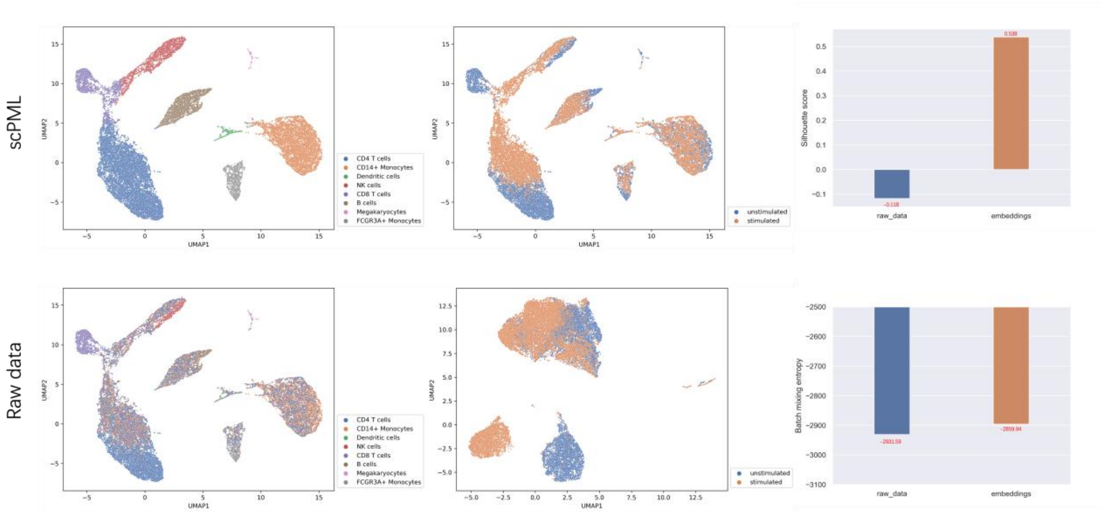

Supplementary Figure 22. UMAP projections and quantitative metrics of scPML and raw data for cross-perturbation experiments. **a**, UMAP projections of scPML and raw data. The first column are the UMAP projections of cells grouped by cell types. The second column show the cells grouped by sample.**b,** Silhouette score and batch mixing entropy of scPML and raw data for cross-perturbation experiment.

Supplementary Note 9: Time complexity analysis

We conducted random stratified sampling on the data, extracting 3000, 5000, 10000, 20000, 30000, and 40000 cell data samples. Firstly, we compared the running time of scPML and Seurat when the training dataset size varied while keeping the test dataset size fixed. As shown in Supplementary Figure 23 a, scPML exhibits linear time complexity and slightly higher running time compared to Seurat. Next, we compared the scenario where the training dataset size remained constant, but the test dataset size increased, as depicted in Supplementary Figure 23 b. In this case, Seurat required more time due to the need for retraining. The average training duration for scPML is 60 seconds, but since the training dataset size remained constant, we did not include training duration in Supplementary Figure 23 b. scPML's prediction time on the continuously growing test set is linear and extremely short. For instance, when the test dataset contained 40000 cells, scPML achieved a prediction running time of 3.43 seconds. This ability to rapidly predict new data makes scPML well-suited for practical prediction tasks.

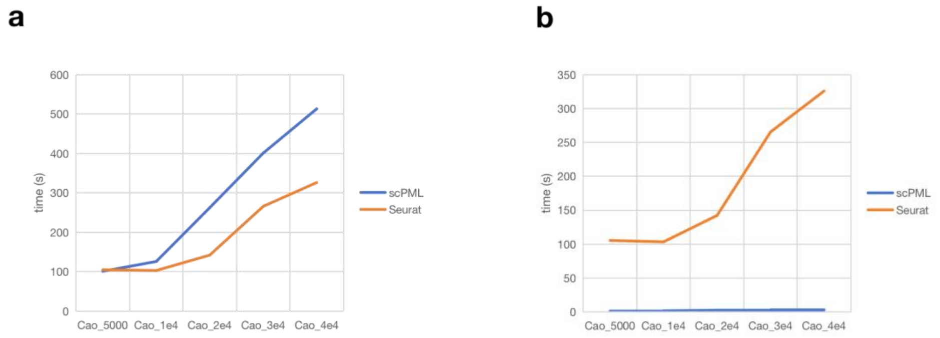

Supplementary Figure 23. Comparison of scPML and Seurat for running time. **a,** Comparison of scPML and Seurat for running time when the training dataset size increases from 5000 to 40000 while keeping the test dataset size constant. **b,** Comparison of the running time of scPML and Seurat when the training dataset size remains constant, but the test dataset size increases from 5000 to 40000.

Supplementary References

1. Abdelaal, Tamim, et al. "A comparison of automatic cell identification methods for single-cell RNA sequencing data." *Genome biology* 20 (2019): 1-19.
2. Baron, Maayan, et al. "A single-cell transcriptomic map of the human and mouse pancreas reveals inter-and intra-cell population structure." *Cell systems* 3.4 (2016): 346-360.
3. Xin, Yurong, et al. "Single-cell RNAseq reveals that pancreatic β-cells from very old male mice have a young gene signature." *Endocrinology* 157.9 (2016): 3431-3438.
4. Segerstolpe, Åsa, et al. "Single-cell transcriptome profiling of human pancreatic islets in health and type 2 diabetes." *Cell metabolism* 24.4 (2016): 593-607.
5. Muraro, Mauro J., et al. "A single-cell transcriptome atlas of the human pancreas." *Cell systems* 3.4 (2016): 385-394.
6. Tirosh, Itay, et al. "Dissecting the multicellular ecosystem of metastatic melanoma by single-cell RNA-seq." *Science* 352.6282 (2016): 189-196.
7. Paulson, K. G., et al. "Acquired cancer resistance to combination immunotherapy from transcriptional loss of class I HLA." *Nature communications* 9.1 (2018): 3868.
8. Song, Qianqian, Jing Su, and Wei Zhang. "scGCN is a graph convolutional networks algorithm for knowledge transfer in single cell omics." Nature communications 12.1 (2021): 3826.
9. Stuart, Tim, et al. "Comprehensive integration of single-cell data." Cell 177.7 (2019): 1888-1902.
10. Aran, Dvir, et al. "Reference-based analysis of lung single-cell sequencing reveals a transitional profibrotic Macrophage." Nature immunology 20.2 (2019): 163-172.
11. De Kanter, Jurrian K., et al. "CHETAH: a selective, hierarchical cell type identification method for single-cell RNA sequencing." Nucleic acids research 47.16 (2019): e95-e95.

12. Kiselev, Vladimir Yu, Andrew Yiu, and Martin Hemberg. "scmap: projection of single-cell RNA-seq data across data sets." Nature methods 15.5 (2018): 359-362.
13. Kanehisa, Minoru, et al. "KEGG: new perspectives on genomes, pathways, diseases and drugs." Nucleic acids research 45.D1 (2017): D353-D361.
14. Fabregat, Antonio, et al. "The reactome pathway knowledgebase." Nucleic acids research 46.D1 (2018): D649-D655.
15. Slenter, Denise N., et al. "WikiPathways: a multifaceted pathway database bridging metabolomics to other omics research." Nucleic acids research 46.D1 (2018): D661-D667.
16. Ji, Zhicheng, and Hongkai Ji. "TSCAN: Pseudo-time reconstruction and evaluation in single-cell RNA-seq analysis." Nucleic acids research 44.13 (2016): e117-e117.
17. Zhang, Chenxing, et al. "Improving single-cell RNA-seq clustering by integrating pathways." Briefings in Bioinformatics 22.6 (2021): bbab147.
18. Cao, Junyue, et al. "A human cell atlas of fetal gene expression." Science 370.6518 (2020): eaba7721.
