# Supplementary material for: scPML: pathway-based multi-view learning for cell type annotation from single-cell RNA-seq data
Source: Commun Biol. 2023 Dec 14;6:1268. doi: 10.1038/s42003-023-05634-z (PMC10721875; doi:10.1038/s42003-023-05634-z)
Supplement: Supplementary file 2 — Description of Additional Supplementary Files [file 42003_2023_5634_MOESM2_ESM.pdf]

Description of Additional Supplementary Files

File name: Supplementary Data

Description: The source data behind the graphs.
