# Supplementary material for: scPML: pathway-based multi-view learning for cell type annotation from single-cell RNA-seq data
Source: Commun Biol. 2023 Dec 14;6:1268. doi: 10.1038/s42003-023-05634-z (PMC10721875; doi:10.1038/s42003-023-05634-z)
Supplement: Supplementary file 4 — Reporting Summary [file 42003_2023_5634_MOESM4_ESM.pdf]

nature portfolio

Corresponding author(s): Yu-An Huang

Last updated by author(s): Nov 13, 2023

Reporting Summary

Nature Portfolio wishes to improve the reproducibility of the work that we publish. This form provides structure for consistency and transparency in reporting. For further information on Nature Portfolio policies, see our Editorial Policies and the Editorial Policy Checklist.

Statistics

For all statistical analyses, confirm that the following items are present in the figure legend, table legend, main text, or Methods section.

n/a	Confirmed	
☐	☒	The exact sample size (*n*) for each experimental group/condition, given as a discrete number and unit of measurement
☐	☒	A statement on whether measurements were taken from distinct samples or whether the same sample was measured repeatedly
☒	☐	The statistical test(s) used AND whether they are one- or two-sided<br>*Only common tests should be described solely by name; describe more complex techniques in the Methods section.*
☒	☐	A description of all covariates tested
☒	☐	A description of any assumptions or corrections, such as tests of normality and adjustment for multiple comparisons
☒	☐	A full description of the statistical parameters including central tendency (e.g. means) or other basic estimates (e.g. regression coefficient) AND variation (e.g. standard deviation) or associated estimates of uncertainty (e.g. confidence intervals)
☒	☐	For null hypothesis testing, the test statistic (e.g. *F*, *t*, *r*) with confidence intervals, effect sizes, degrees of freedom and *P* value noted<br>*Give P values as exact values whenever suitable.*
☒	☐	For Bayesian analysis, information on the choice of priors and Markov chain Monte Carlo settings
☒	☐	For hierarchical and complex designs, identification of the appropriate level for tests and full reporting of outcomes
☒	☐	Estimates of effect sizes (e.g. Cohen's *d*, Pearson's *r*), indicating how they were calculated

Our web collection on statistics for biologists contains articles on many of the points above.

Software and code

Policy information about availability of computer code

Data collection	seurat==4.1.0
Data analysis	Custom software: https://github.com/Kevis9/Cell_Classification<br>Public softwares: scikit-learn == 1.0.2 numpy==1.23.3 pytorch==1.10.2 torch-geometric==2.0.3 networkx==2.8.4 pandas==1.4.3 scipy==1.9.1

For manuscripts utilizing custom algorithms or software that are central to the research but not yet described in published literature, software must be made available to editors and reviewers. We strongly encourage code deposition in a community repository (e.g. GitHub). See the Nature Portfolio guidelines for submitting code & software for further information.

Data

Policy information about availability of data

All manuscripts must include a data availability statement. This statement should provide the following information, where applicable:

- Accession codes, unique identifiers, or web links for publicly available datasets
- A description of any restrictions on data availability
- For clinical datasets or third party data, please ensure that the statement adheres to our policy

All datasets analyzed in the current study are publicly available and can be downloaded from their public accessions. The PBMC data of six different sequencing protocols are available from the Broad Institute Single Cell portal(https: //portals.broadinstitute.org/single cell/study/ SCP424/single-cell-comparisonpbmc-

data).The published pancreatic datasets were downloaded from scRNA-seq data(Baron, Xin, Muraro, Segerstolpe). The source data of mouse liver were downloaded from https: //bis.zju.edu.cn/MCA/. The source data of tumor were downloaded https://www.ncbi.nlm. nih.gov/geo/(GSE72056, GSE10332, GSE118056, GSE117988)

Research involving human participants, their data, or biological material

Policy information about studies with human participants or human data. See also policy information about sex, gender (identity/presentation), and sexual orientation and race, ethnicity and racism.

Reporting on sex and gender	N/A. Didn't perform any sex- or gender-based analyses
Reporting on race, ethnicity, or other socially relevant groupings	N/A. Didn't perform any race- or other socially relevant groupings-based analyses.
Population characteristics	N/A. Didn't conduct any population based analyses.
Recruitment	Used publically available data. Didn't recruit any participants
Ethics oversight	Used publically available data. No study protocol approval is needed.

Note that full information on the approval of the study protocol must also be provided in the manuscript.

Field-specific reporting

Please select the one below that is the best fit for your research. If you are not sure, read the appropriate sections before making your selection.

☒ Life sciences ☐ Behavioural & social sciences ☐ Ecological, evolutionary & environmental sciences

For a reference copy of the document with all sections, see nature.com/documents/nr-reporting-summary-flat.pdf

Life sciences study design

All studies must disclose on these points even when the disclosure is negative.

Sample size	In this work, we investigated the model performance on fifteen datasets: (1) PBMCs: CEL-Seq2 dataset contains 526 cells. (2) PBMCs: 10x Chromium (v3) dataset contains 3222 cells. (3) PBMCs: Drop-seq dataset contains 6584 cells. (4)PBMCs: inDrop dataset contains 6584 cells. (5)PBMCs: Seq-Well contains 3773 cells. (6) PBMCs: Smart-seq2 dataset contains 526 cells. (7) Baron: mouse dataset contains 1886 cells. (8) Baron:human dataset contains 8569 cells. (9) Xin dataset contains 1449 cells. (10) Segerstolpe dataset contains 2133 cells. (11) Muraro dataset contains 2122 cells. (12) GSE72056 dataset contains 3280 cells. (13) GSE10332 dataset contains 4570 cells. (14) GSE118056 dataset contains 6042 cells. (15) GSE117988 dataset contains 10082 cells.
Data exclusions	No data was excluded from the analysis.
Replication	We use average values from different test sets to draw the boxplots. We use different combinations of training datasets and test datasets in cross-species, cross-platforms and multi-view learning experiments.
Randomization	For self-supervised GCN learning, we randomly mask the gene expression of non-zero values.
Blinding	For all the experiments, the model are trained only based on the training datasets with cell types labels. The cell type labels of test datasets are unseen.

Reporting for specific materials, systems and methods

We require information from authors about some types of materials, experimental systems and methods used in many studies. Here, indicate whether each material, system or method listed is relevant to your study. If you are not sure if a list item applies to your research, read the appropriate section before selecting a response.

Materials & experimental systems

n/a	Involved in the study
☒	☐ Antibodies
☒	☐ Eukaryotic cell lines
☒	☐ Palaeontology and archaeology
☒	☐ Animals and other organisms
☒	☐ Clinical data
☒	☐ Dual use research of concern
☒	☐ Plants

Methods

n/a	Involved in the study
☒	☐ ChIP-seq
☒	☐ Flow cytometry
☒	☐ MRI-based neuroimaging
